# Eutrophication history and organic carbon burial rate recorded in sediment cores from the Mar Piccolo of Taranto (Italy)

**DOI:** 10.1007/s11356-023-26317-6

**Published:** 2023-03-16

**Authors:** Janusz Dominik, Simone Leoni, Daniele Cassin, Irene Guarneri, Luca Giorgio Bellucci, Roberto Zonta

**Affiliations:** 1 Istituto di Scienze Marine, Consiglio Nazionale delle Ricerche (ISMAR-CNR), Castello 2737/F, 30122 Venezia, Italy; 2 Istituto di Scienze Marine, Consiglio Nazionale delle Ricerche (ISMAR-CNR), Via Gobetti 101, 40129 Bologna, Italy

**Keywords:** Lagoon, Sediment, Organic carbon, Burial rate, Total nitrogen, Eutrophication proxies, Computed tomography

## Abstract

During the second half of the twentieth century, coastal lagoons in densely populated regions experienced eutrophication due to excessive nutrient inputs. Detrimental effects, including hypoxia/anoxia and harmful algae blooms, have occurred in many Mediterranean lagoons, but their trophic evolution is poorly documented. The lack of adequate monitoring data can partly be offset by examining sedimentary records. In the Mar Piccolo, a lagoon comprising two basins near Taranto (Italy), eutrophication has followed population growth and pollution resulting from naval activities and massive industrialisation. Based on ^210^Pb-dated sediment cores, continuous in situ density profiles obtained with computed tomography, organic carbon (OC) and total nitrogen (TN) content and OC and TN isotopic signatures, this paper reconstructs the history of eutrophication, discusses the sources of organic matter and provides an estimate of the OC burial rate before and during the eutrophic period. OC burial increased in the period 1928–1935 and peaked in the decade 1960–1970. OC and TN content were still high in the surface sediments collected in 2013, despite partial diversion of sewage outfalls in the period 2000–2005. The divergent δ^13^C and δ^15^N signatures of the two basins during the eutrophic period suggest they were affected by different nutrient sources. The OC burial rate during the eutrophic phase (≈ 46 g m^−2^ y^−1^) was close to the world median value for lagoon sediments, and was about twice the burial rate recorded in the preceding oligotrophic phase.

**Supplementary Information:**

The online version contains supplementary material available at 10.1007/s11356-023-26317-6.

## Introduction

Marine near-shore sediments are important global carbon sinks (Atwood et al. 2020; Wilkinson et al. 2018). A proper estimate of past and present carbon burial rates depends heavily on knowledge of coastal sediment accumulation rates on centennial time scales. During the twentieth century, coastal lagoons in densely populated areas became highly productive, as they were fertilised with bioavailable phosphorus and nitrogen from urban agglomerations and cultivated fields. Estimates of external nutrient loading to coastal bays and lagoons are in the same range as deep estuaries (McGlathery et al. 2007), causing eutrophication with subsequent seagrass loss, harmful algal blooms and periodic hypoxia or anoxia. Organic matter burial and degradation rates in sediments can also be modified (McGlathery et al. 2007). Where historical data on the centennial scale are scarce or non-existent, trophic evolution can be inferred from dated sediment cores (Voss et al. 2000; Vaalgamaa et al. 2013 and references therein; Jia et al. 2012) using various proxies for past eutrophication and hypoxia events, as reviewed by Gooday et al. (2009).

Overall trends are relatively well preserved once organic matter (OM) is buried in sediments (Meyers 1994; Lamb et al. 2006), although selective mineralisation of its labile fraction may slightly modify the isotopic signature and C/N ratio. Since the early research on the isotopic signature of OM in shelf sediments (e.g. Peters et al. 1978), many studies have demonstrated its general validity for tracing the origin of sediment organic matter (SOM), although local sources or local anthropogenic perturbations require careful consideration (Vaalgamaa et al. 2013).

Eutrophication can be expected to increase the δ^13^C values of SOM, due to enhanced in situ production of marine phytoplankton, and may also result in increased δ^15^N due to the higher contribution of ammonium nitrogen regenerated from sediments (Gooday et al. 2009). Among other proxies, the Mn/Fe oxide ratio, magnetic susceptibility, diatoms, foraminifers, ostracods and phytoplankton pigments have all been used in lake and/or coastal marine sediments for the assessment of past hypoxic/anoxic conditions in bottom waters (Feuillade et al. 1995; Gibbs-Eggar et al. 1999; Loizeau et al. 2001; Cronin and Vann 2003; Rabalais et al. 2007).

The Mar Piccolo (Taranto, Italy) experienced progressive eutrophication during the twentieth century, but very few water and sediment quality data were recorded before the mid-1980s, although some early studies cited by Caroppo and Portacci (2017, appendix A) provide valuable observations. The Mar Piccolo is a Long-Term Ecosystem Research site (Morabito et al. 2018). Since 1991, four stations have been seasonally monitored, collecting phyto- and zoobenthos samples and measuring the main environmental variables (temperature, salinity, pH, O_2_). Signs of oligotrophication have been observed in recent decades, although harmful algal blooms and anoxia crises persist (Caroppo et al. 2016). Concomitant pollution by metals and toxic organic compounds have also contributed to the degradation of ecosystem functioning (Cibic et al. 2016). Severely impacted by industrial, urban and agricultural pollution, the Mar Piccolo has become the object of numerous environmental surveys, technical reports (Storelli and Marcotrigiano 2000; Cardellicchio et al. 2006; Petronio et al. 2012; ARPA Puglia and ISPRA 2015) and pluri-disciplinary studies, particularly over the last two decades (Cotecchia et al. 2021). A number of important scientific papers appeared in a special issue of *Environ. Sci. Pollut. Res.* (for an overview, see Cardellicchio et al. 2016).

Here, we add a historical perspective regarding the anthropogenic impact on trophic changes in the Mar Piccolo based on the record from sediment cores. Specifically, we examine organic carbon (OC) and total nitrogen (TN) concentrations and their isotopic fingerprints (δ^13^C, δ^15^N) in sediment profiles, in an attempt to detect changes related to trophic evolution from the preindustrial period to the first decade of the twenty-first century. Computed tomography (CT) images and associated CT numbers were used to inspect sediment structure and calculate in situ dry density, which enabled the estimation of the sediment mass accumulation rate (MAR) by the ^210^Pb method. Finally, we estimate the specific burial rate of OC in sediments deposited during the eutrophic and oligotrophic periods.

## Study site

**Fig. 1:**
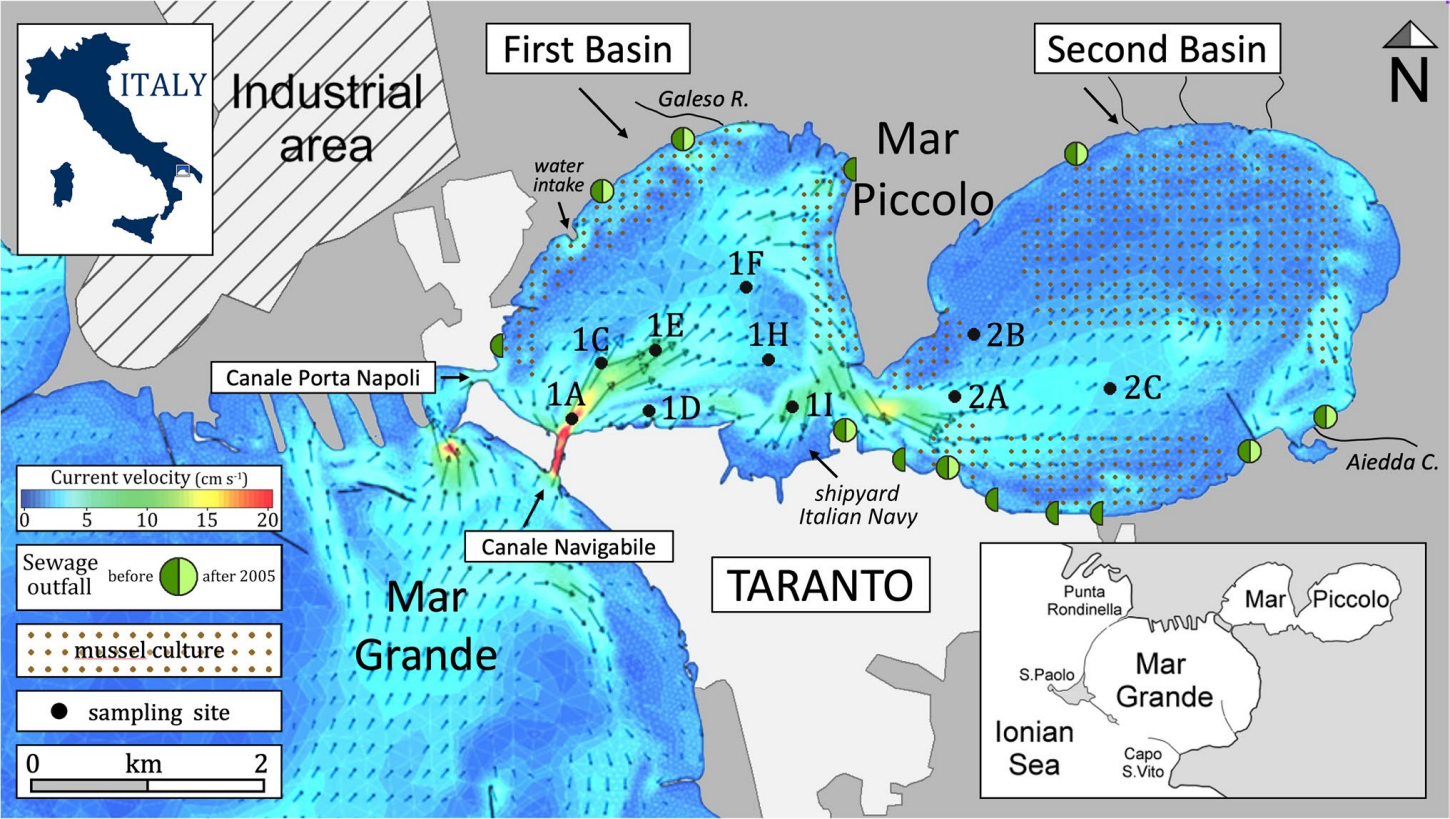
Map of the Mar Piccolo, showing the location of cores, bottom current field (after De Pascalis et al. 2016), mussel-growing areas and sewage outfalls (past and still active after 2005) according to Caroppo et al. (2016). Sampling site nomenclature follows that of the RITMARE project (Cardellicchio et al. 2016)

The Mar Piccolo (surface area ca. 20.7 km^2^) is located northeast of the city of Taranto (Fig. 1). It is a nearly enclosed waterbody with lagoon features (Cardellicchio et al. 2016), divided by rocky promontories into two parts: the 1^st^ basin (*Primo Seno*: western side, maximum depth 13 m) and the 2^nd^ basin (*Secondo Seno*: eastern side, maximum depth 10 m). The 1^st^ basin is connected to the Mar Grande, a bay of the Mediterranean Sea, through two channels (*Porta Napoli* and *Navigabile*). The latter channel was excavated in 1883–1886, enlarging and deepening the ancient moat of the Aragonese Castle, in order to allow the transit of large naval units between the Mar Piccolo and the Mar Grande (Messina 1888).

Tidal range does not exceed 30–40 cm. Water salinity (mean 37 PSU, De Pascalis et al. 2016) is influenced by the presence of about 30 freshwater submarine springs (locally called “*citri*”) and the outfalls of small tributaries (Zuffianò et al. 2016 and references within), the most important of which are the *Galeso River* in the 1^st^ basin (mean discharge 0.52 m^3^s^−1^) and the *Aiedda Canal* in the 2^nd^ basin (0.70 m^3^s^−1^) (De Pascalis et al. 2016). Vertical water stratification occurs, especially during summer. A large cooling water intake system, installed by the ILVA steelworks in the 1960s, draws about 34 m^3^/s of water from the 1^st^ basin for industrial cooling. Bottom current patterns (Fig. 1) show the main stream entering the 1^st^ basin through the *Canale Navigabile*, flowing NE and then into the 2^nd^ basin (De Serio et al. 2018; De Pascalis et al. 2016). According to the numerical model, the mean yearly water renewal times are about 25 and 35 days for the 1^st^ and the 2^nd^ basin respectively (De Pascalis et al. 2016).

In addition to the contribution from industrial plants, anthropogenic impacts are widely distributed within the study area (Calace et al. 2008). Small rivers and *citri* drain the surrounding agricultural soils, and urban sewage from the city of Taranto and nearby towns is discharged into the Mar Piccolo through a number of pipes (Fig. 1). What was once Italy’s most important Naval base, of which only the dry docks are still present, was located on the southern shores of the 1^st^ basin (Cardellicchio et al. 2016).

Since the end of the eighteenth century, the Mar Piccolo has been an important site for shellfish farming (Caroppo and Portacci 2017). Mussel production increased from less than 5 × 10^3^ t y^−1^ prior to 1980 to a maximum of ca. 6 × 10^4^ t y^−1^ in 2005–2006 (higher than any other site in Italy) and then decreased to ca. 4 × 10^4^ t y^−1^ in 2010. During the period 2002–2004, mussel farms covered 26% and 66% of the total area of the 1^st^ and 2^nd^ basins respectively (Caroppo et al. 2012).

## Methods

### Sampling

Sediment cores were collected in June 2013 from 10 sites (Fig. 1) with an SW 104 gravity corer (Carmacoring, Italy), which preserves the sediment–water interface (Bellucci et al. 2012). The length of the cores ranged from 97.8 cm (site 1D) to 135.8 cm (site 2A), while the diameter was 110 mm. The cores were maintained at 4 °C in the PVC liner used for sampling.

### Magnetic susceptibility

The cores were scanned for whole-core magnetic susceptibility (χ) soon after sampling with a Bartington MS2 system equipped with a 120-mm loop sensor at a sampling interval of 1 cm every 10 s. Background values were estimated by taking repeated measurements in air before and after the analysis.

### CT-scan analysis

Sediment cores were examined by X-ray computed tomography (CT-scan) at a 1-mm down-core resolution using a Toshiba Aquilion 64 (voltage = 100–130 kVp, dose for scan = 320–370 mA). The CT-scan data were processed with Horos v2.0.2 software (Horos Project). Tomographic intensity profiles measured along the core (CT number expressed in HU units on the Hounsfield scale) were obtained by means of algorithms (MIP, AIP, MinIP), as described in Zonta et al. (2021).

### Sub-sampling of cores and determination of water content and in situ dry density

The PVC liners were split lengthwise, and one-half of each sediment core was subsampled by selecting 2-cm-thick layers along the depth profile, chosen on the basis of CT images. After removing shells and other large debris, the samples were thoroughly homogenised. Water content (*W*_*c*_) was determined from an aliquot of a sample dried in an oven at 105 °C until it reached a constant weight (Percival and Lindsay 1997).

The in situ dry density of the sediment (*ρ*^*s*^) was calculated from the *W*_*c*_ and the specific sediment particle density, the estimate of which took account of variations in SOM and CaCO_3_ content. It was assumed that inorganic carbon (IC) was bound as CaCO_3_ (calcite and aragonite). The mean CT numbers, measured using the average intensity projection (AIP) setting, were used to obtain continuous *ρ*^*s*^ profiles, as described in detail by Zonta et al. (2021). Here, we used raw AIP values, as opposed to values from the selected region of interest (ROI), to calculate mean *ρ*^*s*^ including fragments of shells and voids (Text S1 in Supplementary Information).

### Organic carbon, total nitrogen, inorganic carbon and stable isotopes

Total nitrogen (TN) and total carbon (TC) were determined on duplicate samples using a ThermoFisher Flash 2000 IRMS Elemental Analyser (EA). Subsamples for OC determination were first decarbonated with HCl 1.5N. Inorganic carbon (IC) was calculated as TC minus OC. Stable isotopic analyses of OC and TN were carried out using a FINNIGAN Delta Plus mass spectrometer, which was directly coupled to the EA by means of a CONFLO interface for continuous flow measurements (Tesi et al. 2007). The average standard deviation of each measurement, based on replicate analyses of the same sample, was ± 0.07% for OC and ± 0.009% for TN. All isotopic compositions are presented in the conventional *δ* notation and reported as parts per thousand (‰). For determination of δ^13^C, the IAEA reference sample IAEA-CH7 (polyethylene, − 32.15‰ vs. VPDB) was used for calibration of the mass spectrometer. Uncertainties, as determined from routine replicate measurements, were lower than ± 0.05‰. The internal standard for isotopic measurement of TN was IAEA-N-1 (ammonium sulphate, + 0.4‰ vs. air). Errors derived from replicate analyses of the standards were ± 0.2‰.

### Grain-size analysis

Measurement was performed with a Microtrac FRA-9320 X100 laser diffraction particle size analyser (Leeds & Northrup, USA) on approximately 2 g of wet sediment dispersed in distilled water, yielding volumetric percentages of particles in 50 size classes in the range 0.1–700 μm.

### Determination of sedimentation rates by the ^210^Pb method

^210^Pb determination was performed on four cores (1A, 1F, 1I, 2C) using alpha ^210^Po spectrometry, assuming secular equilibrium between the two radionuclides. ^210^Po extraction and measurement procedures are described by Bellucci et al. (2007). ^210^Pb activity supported by ^226^Ra (^210^Pb_sup_) was estimated from total ^210^Pb activity measured in the deep part of the sediments, where it remained approximately constant. These values were in good agreement with ^226^Ra activity reported by Guzzi et al. (2009) for the sediments of the Mar Piccolo. In each of the four cores, ^210^Pb activity was determined in 5 to 8 samples 2 cm thick, distributed over depth intervals ranging from 0–28 to 0–44 cm. MARs expressed in g cm^−2^ y^−1^ were determined using the constant ^210^Pb_ex_ flux and constant sedimentation rate (CF-CS) model (Krishnaswamy et al. 1971; Appleby and Oldfield 1978). The corresponding mean sediment accumulation rates (SARs) expressed in cm y^−1^ were then calculated. It appeared that ^210^Pb_ex_ distribution with depth was less consistent in two cores (not shown), because of selective erosion (winnowing) of sediments in the proximity of the *Canale Navigabile* (core 1A) or perturbation of sediments by resuspension or displacement (core 1I). These two cores were considered unsuitable for reliable time scale determination.

### Statistics

As some variables were not normally distributed (according to the Shapiro–Wilk test), box and whisker plots were used for graphical presentation of the data and a Mann–Whitney test was used to assess significant differences between groups at *p* = 0.05. Spearman rank correlation was used to assess relations between variables. Statistics were calculated with Microsoft Excel for Mac v. 14.6.0., StatPlus:mac v6.1.55 AnalystSoft.Inc and Real Statistics (Zaiontz 2021).

## Results and discussion

In order to reconstruct the eutrophication history from the sedimentary record, *ρ*^*s*^ was first determined from the *W*_*c*_ and its relation to the CT number (‘CT profiles and their relation to in situ dry density’) was assessed. Time scales for one core from each basin were constructed based on the sediment MAR determined by the ^210^Pb method (‘Sediment accumulation rates’). After presentation and discussion of the profiles of relevant variables in the two dated cores and two others from the 2^nd^ basin (‘Reconstruction of eutrophication history’), we examined the sediment characteristics and proxies’ profiles and statistics in all 10 cores in order to extend the observations to the whole of the Mar Piccolo (‘Characteristics of sediments from the Mar Piccolo as a whole’). Finally, we estimated the OC burial rate in the eutrophic period and the preceding oligotrophic period (‘Estimation of the OC burial rate’).

### CT profiles and their relation to in situ dry density

The CT number essentially depends on the bulk sediment density, which is proportional to both porosity and particle density. Regression of the CT number against *ρ*^*s*^ (see Fig. S1.1 in Supplementary Information) showed a high determination coefficient (*R*^2^ = 0.93). Using the regression equation, *ρ*^*s*^ variations with depth were calculated for the upper 50 cm of the sediment cores with a resolution of 2 cm (see Fig. S1.2 in Supplementary Information for cores from the 1^st^ basin), enabling calculation of the MAR. CT images provided information on the presence of high-density objects (e.g. shells and shell fragments), compact layers and low-density volumes (e.g. gas bubbles or burrows). Low-density sediment sections had higher *W*_*c*_, usually corresponding to sediments rich in OC and TN (Zonta et al. 2021).

### Sediment accumulation rates

The two cores (1F and 2C) showed ^210^Pb_ex_ activity decreasing approximately exponentially with cumulative sediment weight, which enabled estimation of the MAR with the CF-CS model (see Fig. S2 in Supplementary Information). In core 1F, the MAR was calculated to be 0.15 ± 0.01 g cm^−2^ y^−1^ with minimal uncertainty (determination coefficient *R*^2^ = 0.99). This rate corresponds to the mean SAR of 0.25 cm y^−1^. The determination of the MAR was less straightforward in core 2C, because of the uncertainty of ^210^Pb_sup_ activity. We assumed that ^210^Pb_sup_ was equal to the mean value of two samples at depths of 33 and 36 cm with very similar ^210^Pb activity, although the sample at 44–46 cm showed lower activity. At the latter depth, however, the sediment was more compact, with a coarser grain size and less OC. These features, combined with CT images, suggested that the 44–46-cm layer was formed of a coarser terrigenous material. The resulting MAR in this core was 0.17 ± 0.04 g cm^–2^ y^–1^, corresponding to a mean SAR of 0.36 cm y^–1^.

The SARs obtained for cores 1F and 2C were similar to what was reported by Guzzi et al. (2009) for three cores collected in the 1^st^ basin (0.28, 0.30 and 0.38 cm y^–1^), implying a fairly homogenous rate of recent sediment deposition in a large area of the Mar Piccolo.

### Reconstruction of eutrophication history

The time scale assigned to each core enabled temporal interpretation of CT images, changes in nutrient content and calculation of OC burial fluxes. It also shed light on the evolution of the SOM’s isotopic signature over time.

#### Chronology and fluxes

**Fig. 2:**
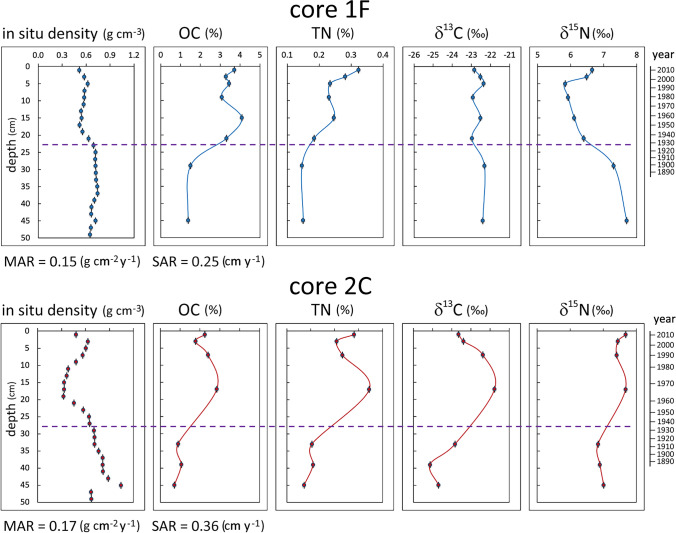
Depth profiles for in-situ dry density (*ρ*^*s*^), nutrient element content and related isotopic signatures (0–50 cm depth) in dated cores from 1^st^ basin (core 1F) and 2^nd^ basin (core 2C). For non-sampled intervals, *ρ*^*s*^ values were inferred from CT (AIP) measurements. Time scales are derived from ^210^Pb dating, assuming constant mass accumulation rates (MAR in g cm^−2^ y^−1^). The corresponding mean sediment accumulation rates (SAR, in cm y^−1^) are also given. Dashed horizontal lines depict the probable depth and corresponding date of the onset of eutrophication

The strong OC and TN gradients that appear in core 1F at depths of 22 to 30 cm correspond to the period 1900–1935 (Fig. 2). In situ dry density, which is inversely correlated with both OC (Spearman rank correlation −0.69) and TN (−0.87) content, could be used to estimate changes in SOM content with a better resolution than our discontinuous sampling. Lagoon sediments rich in OM retain more pore water and thus their *ρ*^*s*^ is lower (Zonta et al. 2021). Judging from the *ρ*^*s*^ profile in core 1F, accumulation of OM started increasing around 1928 (dashed line in Fig. 2) and peaked in the 1960s. Similarly, in the other dated core (2C), an increase in OC content by a factor of 3 was observed between 17 and 33 cm. Judging from the *ρ*^*s*^ profile, OC content started to increase at a depth of about 28 cm, around 1935, and peaked between 14 and 20 cm, corresponding to the decade 1960–1970 (Fig. 2).

OC fluxes preserved in the surface sediments (0–2 cm depth) were calculated at 56 and 38 g m^−2^ y^−1^ in cores 1F and 2C respectively. The estimated fluxes of OC preserved in sediments deposited in the 1960s were 69 and 48 g m^−2^ y^−1^ for cores 1F and 2C respectively. For comparison, OC content stored in nineteenth century sediments corresponds to fluxes of 21–39 g m^−2^ y^−1^ at site 1F and 17–19 g m^−2^ y^−1^ at site 2C.

Fluxes calculated from buried sediment can be underestimated due to diagenetic mineralisation of the labile fraction of OM, although OM is usually better preserved in anoxic sediments than in the presence of oxygen (Gooday et al. 2009; Middelburg and Levin 2009). Higher OC and TN content in the surface samples (0–2 cm) than the underlying sample (2–4 cm), observed in both cores, could be due to early diagenesis in the subsurface layer (Fig. 2). Even with some losses due to mineralisation, the high OC concentrations at depths of 15 cm (1F) and 17 cm (2C) indicate higher particulate organic matter (POM) input and/or biomass production and burial during the 1960s than in the first decade of the twenty-first century and much higher than in the nineteenth century. At the beginning of the twentieth century, environmental quality was good, with high biodiversity and the bottom covered by macroalgae and seagrasses (Lo Giudice (1913) cited in Caroppo and Portacci 2017).

**Fig. 3:**
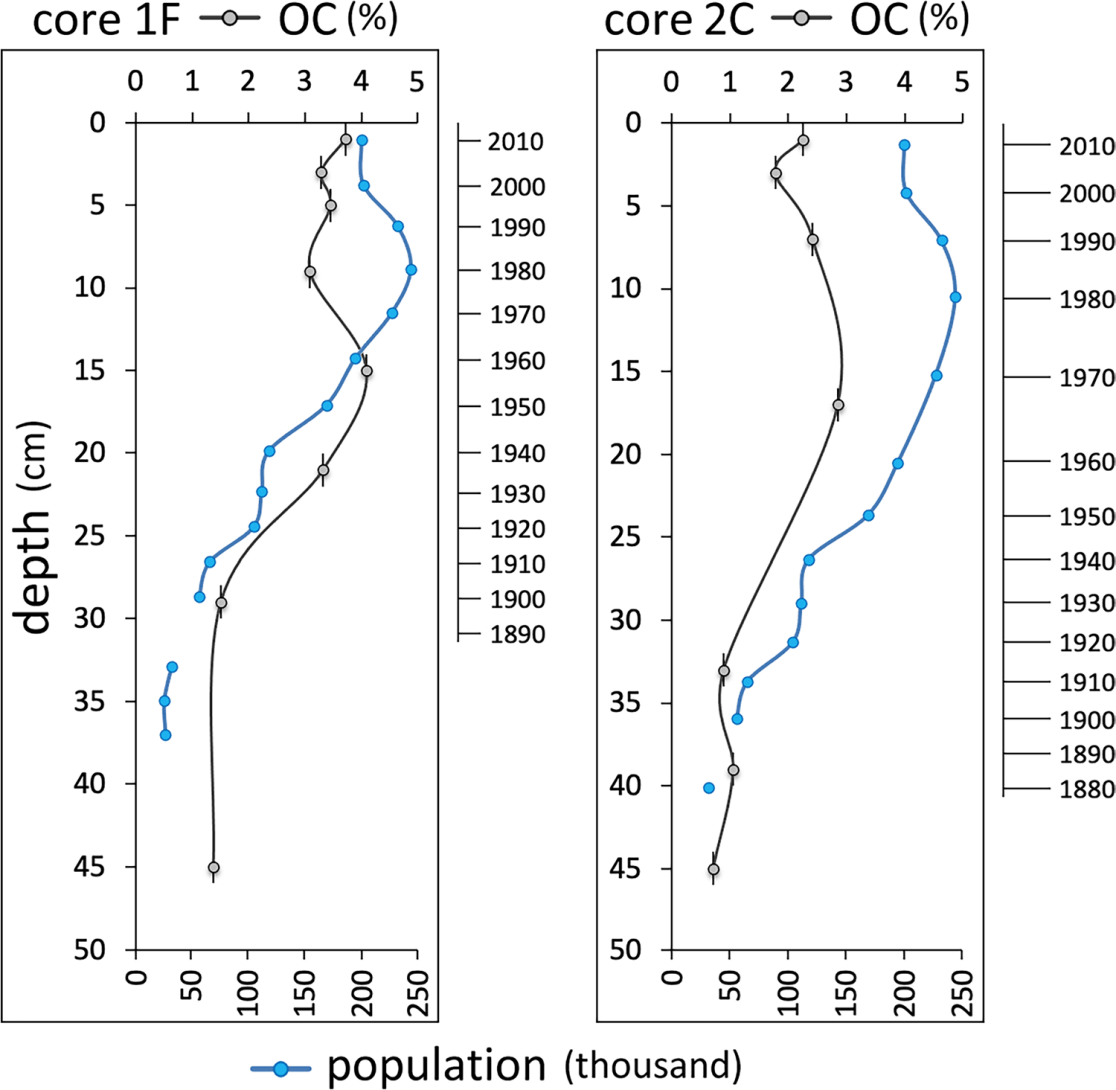
History of population growth in Taranto (ISTAT 2022) compared to sediment organic carbon content in the 1^st^ (core 1F) and 2^nd^ (core 2C) basins

A considerable increase in the OC and TN content of sediments between 1928 and 1970 was probably related to rapid population growth, leading to enhanced nutrient supply in the form of untreated sewage. The population of Taranto increased from the mid-nineteenth century until 1980, while the OC content of sediments stabilised a decade earlier (Fig. 3).

This may imply that factors other than nutrient availability limited primary production or burial of OC (e.g. light, silica content, pollution, mussel production) during the late eutrophic phase. The closure of 6 out of 13 sewage outfalls in the Mar Piccolo in the period 2000–2005 decreased nutrient input by about 25% (Caroppo et al. 2016), but the results could not be detected in the 2-cm-thick surface layer (representing 6–7 years of sedimentation) in the core collected in 2013. Nevertheless, the available measurements of dissolved nitrogen (NH_4_, NO_3_ and NO_2_) and phosphorous (PO_4_) compounds in the surface water of the Mar Piccolo show a decrease in median concentrations by an order of magnitude between the periods 1991–2000 and 2001–2009, with no further changes in the period 2013–2014 (Kralj et al. 2016).

A partial diversion of sewage triggered a change in phytoplankton composition, and between 1991 and 2014 its biomass fell by a factor of 1.7 in the 1^st^ basin but was not affected in the 2^nd^ basin (Caroppo et al. 2016).

Chl-a measurements in 1984–1986 showed mean concentrations between 8.7 and 11.2 μg L^−1^ with peaks up to 22.4 μg L^−1^, while the mean concentrations were much lower in 1990–1992 at about 2.1–2.5 μg L^−1^ (ILVA 2005). Other surveys carried out in various periods between 1991 and 2014 reported relatively low Chl-a mean annual concentrations in the range 1–3 μg L^−1^ (Alabiso et al. 2005; Caroppo et al. 2016; Morabito et al. 2018). Low phytoplankton biomass corresponded to periods of increased mussel growth and vice versa (Caroppo et al. 2012; Caroppo and Giordano 2022). The top-down control of phytoplankton biomass by bivalve grazing implies that bottom-up control of phytoplankton development (e.g. nutrient loading) becomes less important (Prins et al. 1997). This may, at least partially, explain why SOM content did not increase further in the 80^th^ and 90^th^ despite still high nutrient loads from sewage outfalls. Indeed, mussel production was less then 5 × 10^3^ t y^−1^ before 1980, reached 60 × 10^3^ t y^−1^ in 2005–2006 and then declined (Caroppo et al. 2012).

**Fig. 4:**
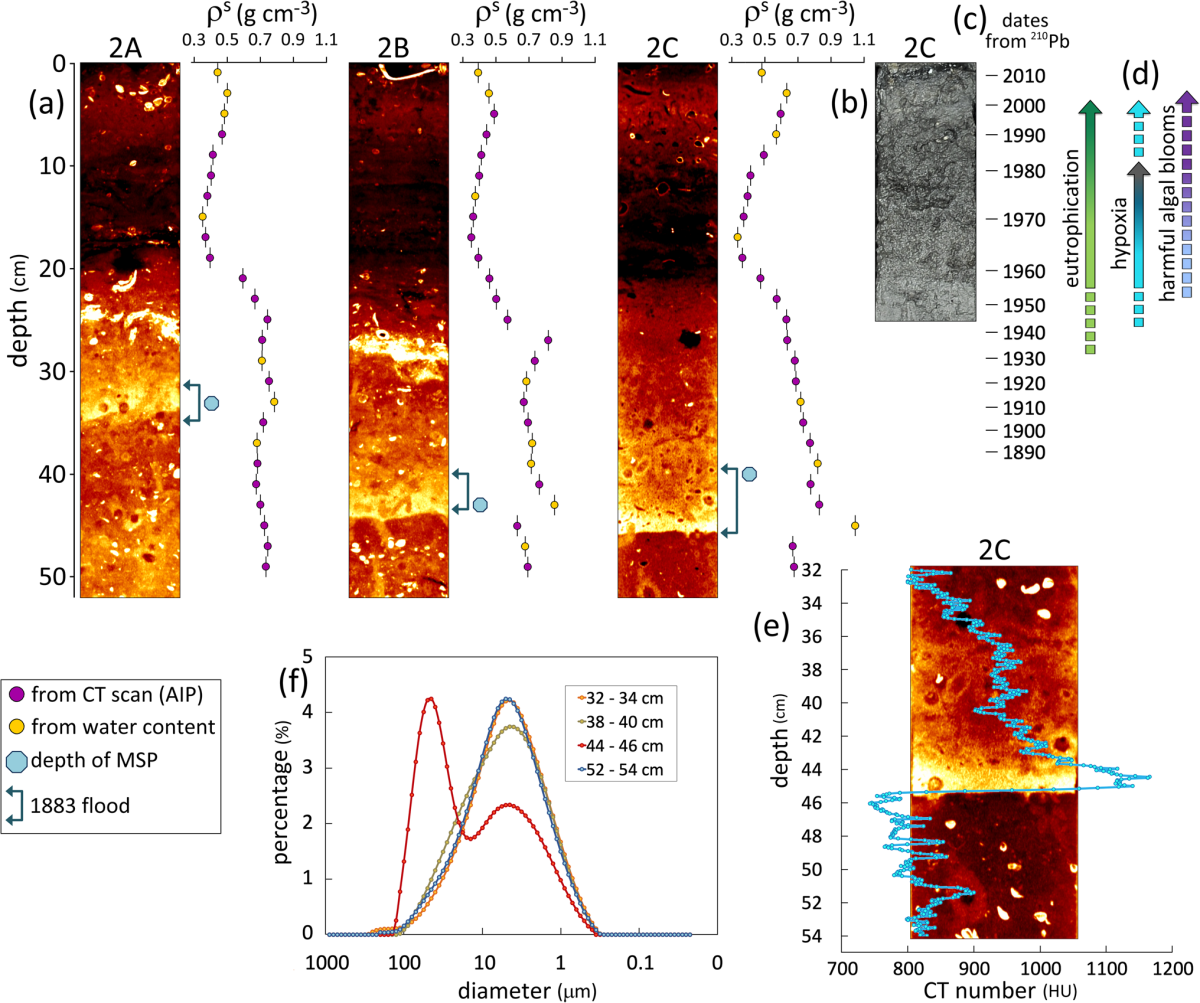
**a** CT images of the upper 50 cm of sediment cores and the corresponding in situ dry density (*ρ*^*s*^) profiles reflecting the development of eutrophication in three cores from the 2^nd^ basin of Mar Piccolo. The dark sections correspond to low density, OM-rich intervals, while the light-coloured intervals correspond to high-density sediments or shell fragments. The depths of the magnetic susceptibility peaks (χ peaks) are depicted with light blue dots and the flood layer sections are marked with arrows. **b** Black and white photograph of the upper section of core 2C cut in half longitudinally showing the abundance of OM with the presence of filamentous algae remains. **c** Time scale for core 2C obtained with the ^210^Pb method. **d** Schematic representation of the evolution of eutrophication and related effects in the 2^nd^ basin, based on our observations and literature data. **e** Detailed CT profile (mean of 4 profiles, in maximum intensity projection (MIP) setting) and enlarged CT image from core 2C revealing density changes to the sedimentary sequence that was formed as a result of the historic flood in 1883. **f** Grain-size spectra of samples collected at four depth intervals with respect to the sedimentary sequence shown in (**e**); the bimodal distribution in the 44–46 cm sample reflects the heterogeneity of the sampling interval (sand and underlying silty clay)

Based on Chl-a concentrations, Alabiso et al. (2005) noted an increasing degree of eutrophication moving from Mar Grande toward the 2^nd^ basin that they attributed to different levels of confinement. Indeed, the dark colours in the CT images of three cores from the 2^nd^ basin (Fig. 4a) at depths of approximately 10–20 cm depict low-density intervals, particularly rich in OM. Remains of filamentous algae were abundant in these intervals, as shown in the black and white photograph of the upper part of core 2C cut longitudinally (Fig. 4b). High OM content, the almost black colour of the sediments and the presence of filamentous algae all suggest eutrophic conditions in the period 1960 to 2000, as indicated by the time scale derived from ^210^Pb dating in core 2C (Fig. 4c). Since 1938, high biomass occasionally occurred, particularly in the 2^nd^ basin, due to harmful algal blooms, mainly dinoflagellates and diatoms, causing anoxia crises and mussel kills (Caroppo et al. 2016 and reference therein) (Fig. 4d). Usually linked to eutrophic conditions, these blooms and related oxygen depletion also occurred after partial sewage diversion (Labianca et al. 2020).

Sedimentary sequence and *ρ*^*s*^ profile in core 2C and two other cores collected in the 2^nd^ basin were very similar on CT images. However, shell-rich horizons were present in cores 2A and 2B at depths of 25 and 27 cm respectively, but such a horizon was missing in core 2C, although some scattered shell fragments were present at depths of 31–35 cm. In this latter core, a high-density sandy layer was present at a depth of 45 cm (Fig. 4a). The CT number suddenly increased from about 735 HU at a depth of 46.0 cm to 1172 HU at 44.5 cm (Fig. 4e). It decreased rapidly to 1020 HU at 43.0 cm and then more gradually to 866 HU at 34.0 cm. Deposited in the deeper part of the 2^nd^ basin, the sedimentary sequence is most probably the result of the historic flood that occurred in 1883.

According to information collected by Mastronuzzi et al. (2013), a major flood took place during the night between 14 and 15^th^ September 1883, when heavy rainfall hit the eastern part of the province of Taranto, causing abundant flooding and runoff with an exceptional sediment load. The waters of the Mar Piccolo rose by up to 2 m above sea level, flooding the surrounding countryside and destroying the *Porta Napoli* bridge over the homonymous channel. It is likely that huge sediment input, possibly from the *Aiedda* channel, induced a turbidity current resulting in the deposition of a sandy layer about 1 cm thick in the deep part of the 2^nd^ basin of Mar Piccolo. This high-density layer was covered by finer sediments deposited from suspension originating from the same flood (34–42 cm) and was finally overlaid by the normal sediment of the lagoon (see detailed CT profile in Fig. 4e and grain-size spectra in Fig. 4f). A χ peak, most probably resulting from the abundance of fine magnetic particles from the catchment soil, was found at a depth of 40 cm. The lowest δ^13^C value (−25.1 ‰), at a depth of 38–40 cm (Fig. 2), also indicates a strong contribution of OM derived from land.

High-density layers accompanied by χ peaks were also detected in cores 2A and 2B at depths of 33 and 43 cm respectively (Fig. 4a), which indicates a correspondence between the three cores. However, no sand layer was found, because these sites are shallower and located in the western part of the 2^nd^ basin, and thus in the area not affected by the turbidity current. In the same layers, δ^13^C values were at least 1‰ lower than in the over- and underlying sediments, also indicating a strong OM contribution from land.

The mean MAR up to the date of the flood calculated from the depth of the χ peak in core 2C was 0.17 g cm^−2^ y^−1^, in perfect agreement with the MAR obtained from ^210^Pb dating. The depths of the χ peaks in cores 2A and 2B, in which no ^210^Pb data were available, yielded mean MARs of 0.14 and 0.19 g cm^−2^ y^−1^ respectively.

The deposition of a sandy layer overlying fine-grained sediments may cause immobilisation of phosphorus and possibly nitrogen compounds, which would normally be remobilised and recycled in the water column, as documented in some eutrophic lakes (Span et al. 1992). This may lower the concentrations of phosphorous and possibly fixed nitrogen in surface waters, and thus temporarily decrease planktonic primary productivity.

#### Isotopic signatures and OM sources

There were some notable differences between cores 1F and 2C in the carbon and nitrogen isotopic signatures (Fig. 2). In core 1F, a marked increase in OC and TN content in the upper section seems to be accompanied by a substantial decrease in δ^15^N, from 7.3 ‰ at a depth of 29 cm to 5.6‰ at a depth of 5 cm, followed by an increase to 6.6‰ in the surface sample. Apart from small fluctuations, there was no significant difference in the δ^13^C signature between the lower (mean −23.0 ± 0.9) and upper (mean − 22.7 ± 0.3) sections. In core 2C, OC and TN content was higher in the upper section, as were both δ^13^C (from −23.8‰ at a depth of 33 cm to −21.8‰ at a depth of 17 cm) and, to a lesser degree, δ^15^N (from 6.8 to 7.7‰). The increase in δ^13^C can be explained by a greater contribution of OM of planktonic or macroalgal origin (δ^13^C usually about −20‰) relative to terrestrial OM (usually <  −25.5‰).

Cibic et al. (2016) and Bongiorni et al. (2016) also noted higher δ^15^N in POM and SOM (at a depth of 0–3 cm) in the 2^nd^ basin than the 1^st^, which they attributed to higher local metabolic activity, greater discharge of treated sewage or N-rich wastes from the extensive mussel farming in the 2^nd^ basin. Using a mixing model, Cibic et al. (2016) estimated the contribution to SOM in surface sediments at site 2C from phytoplankton, macroalgae, terrestrial/riverine POM, treated sewage and lagoon POM to be roughly 20% from each source. For site 1E (1^st^ basin, close to 1F), the contribution from sewage was lower (7%), while the contributions from terrestrial/riverine POM (29%) and phytoplankton (26%) were higher.

The effects of anthropogenic activities on coastal SOM are not always easy to interpret through δ^15^N signals, although increases in the stable nitrogen isotope ratio have often been considered a sign of eutrophication. Many authors have noticed that massive use of manure and chemical fertilisers, which are transformed in the soil*,* raises the δ^15^N of the nitrate delivered by rivers (e.g. Voss et al. 2000). Moreover, δ^15^N increases when the fixed nitrogen derives from regeneration, e.g. NH_4_^+^ (Gooday et al. 2009).

The δ^15^N of dissolved nitrogen in untreated sewage can be more than 10‰, and secondary and tertiary sewage treatment may further increase this value (Savage 2005; Samper-Villarreal 2020 and references within). However, much lower δ^15^N values (−1.1 to + 7.2 ‰) in untreated sewage sludge were reported by van Dover et al. (1992) and in sewage-derived POM (around 3‰ on average) by Tucker et al. (1999). Large quantities of terrestrial POM originating from sewage could potentially explain the δ^15^N-depleted values in SOM accumulating in the 1^st^ basin between 1930 and 1990 (6.4 to 5.8‰) compared to the values in SOM deposited before 1900 (7.3 to 8.5‰), when the contribution of POM from sewage was still low. A similar interpretation was proposed by Vaalgamaa et al. (2013) for ^15^N depletion in coastal sediments receiving untreated sewage in the Töölönlahti Bay in the Baltic Sea. In the Mar Piccolo, several sewage outfalls were present in both basins but no depletion of ^15^N was observed in the 2^nd^ basin (core 2C, Fig. 2). Unless the type or volume of sewage discharged into the two basins was very different, it is difficult to explain the observed difference.

Studies of macrophytes revealed considerable differences in the macroalgal community between the early and late twentieth century. Cecere and Petrocelli (2009) noted the absence of well-structured macroalgal populations and healthy phanerogam meadows in the latter period, which they attribute to heavy anthropogenic pressure. It is not clear whether such evolution impacted the δ^15^N of SOM, but Hong et al. (2019) also reported ^15^N-depleted OM in sediments contaminated with persistent toxic substances introduced by creeks draining the industrialised catchment of Lake Sihwa. The hypothesis of an effect due to increased toxic substance contamination may also be corroborated by the fact that in highly contaminated sediments from the 1^st^ basin of the Mar Piccolo (site 1I), benthic ecosystem functioning, including microbial processes, was greatly inhibited. This may imply “a limited transfer of OC either to a solid microbial loop or to the higher trophic level” (Franzo et al. 2016), and possibly less efficient nitrogen recycling. This may also contribute to a lower δ^15^N in part of the 1^st^ basin than in the less polluted 2^nd^ basin, but the ^15^N-depleted POM input from the untreated industrial sewage system seems to be a more plausible explanation.

### Characteristics of sediments from the Mar Piccolo as a whole

In this section, we examine the sediment characteristics and proxies’ profiles and statistics in all 10 collected cores in order to extend the observations to the whole of the Mar Piccolo.

#### Sediment grain size

Accumulation of OM in coastal sediments depends on sediment texture, with fine sediments being richer in OM. Sediments in the Mar Piccolo were mainly clayey silt with the silt fraction accounting for 60%, followed by clay (30%) and sand (10%). The sand fraction was dominant only in the upper section (0–14 cm) of core 1A, which was recovered in the proximity of the *Canale Navigabile* (Fig. 1), where bottom currents were stronger (De Serio et al. 2018; De Pascalis et al. 2016). The sand fraction was mainly composed of shells and shell fragments and the sediments in this location were poor in OC and TN. For more details on sediment grain size, see Text S4 in Supplementary Information.

#### Magnetic susceptibility as a possible indicator of hypoxia/anoxia

Magnetic susceptibility peaks can potentially be used as a proxy of eutrophication and anoxia (Gooday et al. 2009). In all examined cores, χ peaks of varying intensity were observed (see Fig. S3.1 in Supplementary Information). In the cores from the 1^st^ basin, major χ peaks occurred near the surface at a depth of 3–7 cm except in core 1D (at 15 cm). In three cores (1E, 1H and 1I), minor χ peaks occurred at a depth of 21 cm. Major χ peak intensity varied from 2358 to 793 (10^−6^, SI), decreasing in intensity from NW to SE.

In the cores from the 2^nd^ basin, major χ peaks occurred at depths of 33–45 cm, with intensity ranging from 843 to 280 (10^−6^, SI), and minor peaks (from 171 to 79 10^−6^, SI) occurred near the surface (at depths of 3–5 cm). The former were clearly related to a flood layer (see ‘Chronology and fluxes’). The near-surface χ peaks were one order of magnitude weaker than comparable peaks in the 1^st^ basin.

χ in sediments is caused by the presence of magnetic minerals, which can be of varying natural origin: either (a) allogenic—detrital magnetic minerals from soil in the catchment or from forest/steppe fires, or (b) authigenic—biogenic transformation of trivalent iron in a reducing environment by magnetotactic bacteria (Oldfield 2007, 2013). χ peaks arising from natural magnetite in catchment soil have been used for sediment core correlations (Loizeau et al. 1997; 2003).

The presence of magnetic particles can also result from anthropogenic activity. For example, magnetite spherules from coal combustion can be delivered from the atmosphere near industrial sites (Morris et al. 1994), and enhanced χ has been found in soils near iron production and processing plants (Schmidt et al. 2005). Magnetite can also be formed by dissimilatory reduction of iron originating from sewage treatment plants that use Fe-hydroxide flocculation for phosphate removal (Gibbs-Eggar et al. 1999).

Nearly all of these sources may be important in the Mar Piccolo sediments. In principle, only the occurrence of secondary magnetic Fe-minerals (e.g. greigite) can be considered as an indicator of anoxia (Gooday et al. 2009). Although in the Mar Piccolo near-surface χ peaks occur in sediments formed during the late eutrophic phase, we believe that they are related to industrial activity as their intensity decreases with distance from industrial sites. Magnetic minerals may originate from coal combustion in the nearby steelworks, and a similar decreasing trend in polycyclic aromatic hydrocarbon (PAH) intensity from east to west was observed (see Fig S3.2 in Supplementary Information)*.* A contribution of magnetic minerals from iron ore processing is also likely, as suggested by the presence of magnetic spherules and metallic shards in the sand fraction of the Mar Piccolo sediments (De Marco et al. 2004).

We concluded that χ peaks in the Mar Piccolo sediments cannot be attributed solely to in situ greigite formation and thus be considered as a proxy for hypoxia/anoxia, even though favourable conditions for its formation were certainly present.

#### Temporal and spatial distribution of organic matter in Mar Piccolo sediments

**Fig. 5:**
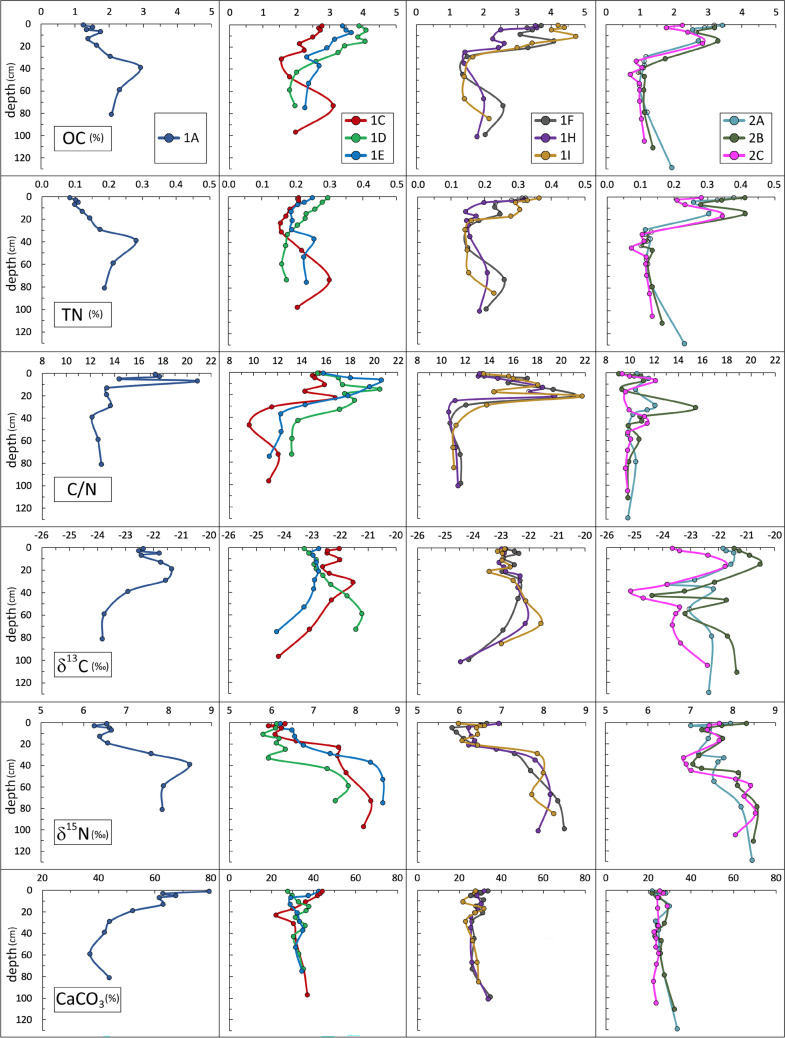
Organic carbon, total nitrogen content, OC/TN ratio, δ^13^C, δ^15^N and CaCO_3_ content in sediment cores from the Mar Piccolo

Profiles with the depths of the examined variables in the 10 sediment cores are shown in four panels (Fig. 5) arranged by geographical position approximately from west to east. Although there were differences between the cores in terms of the variables’ absolute values, the general trends with depth were similar in 6 out of 7 cores from the 1^st^ basin and all 3 cores from the 2^nd^ basin. Minor discrepancies were probably due to differences in sediment accumulation rates or sub-sampling depths.

As a general trend, there were evident differences between the upper and lower sediment sections for the majority of variables. The upper sections were richer in OC and TN, and in the 1^st^ basin they had higher C/N ratios and lower δ^15^N (Fig. 5). The profiles for core 1A, located close to the *Canale Navigabile*, are shown separately (left panels in Fig. 5), as they displayed distinct concentration trends for OC, TN and CaCO_3_. The observed differences were mainly caused by a very high abundance of shell fragments, especially in the upper part of the core. After the modification of the *Canale Navigabile* (1883–1886), stronger currents in this area (De Pascalis et al. 2016; De Serio et al. 2018) probably caused fine-particle winnowing and shell fragment enrichment.

Correlations and descriptive statistics were processed for the following subsets of samples: (1) all cores and depths; (2) upper and lower sections of all cores; and (3) upper and lower sections of cores from each basin separately. The boundary between the upper and the lower sections was determined for each core primarily based on the OC, C/N and δ^15^N profiles, as these variables usually showed clear-cut gradients at depths of 22–38 cm (for the borderline depths, see Table S4.2 in Supplementary Information).

For the whole dataset, highly significant correlation coefficients (Spearman's rank correlations) were found between OC and TN (0.86), and between δ^15^N and the OC/TN ratio (− 0.73). The former was stronger in the lower sections of sediments (0.94) than the upper sections (0.69) and stronger in the 2^nd^ basin (0.94) than the 1^st^ (0.83). This was because the OC/TN ratios in the 2^nd^ basin were similar in the upper and the lower sections, while in the upper section of the 1^st^ basin the ratios were much higher than the lower section (see Table S4.1 in Supplementary Information).

Although observed for the whole dataset, the negative correlation between δ^15^N and OC/TN was clearly seen only in the 1^st^ basin, where the median OC/TN ratio was 16.4 in the upper section and 11.6 in the lower section, while the median δ^15^N was 6.3‰ and 7.9‰, respectively (see Table S4.1 in Supplementary Information). Considering samples from the upper section only or samples from the lower section only, there was no correlation between these variables.

Strong positive correlations were observed between CaCO_3_ content and median particle size (D50) and between CaCO_3_ content and the sand fraction for the whole dataset and for the upper and lower sections of the 1^st^ basin, due to its higher abundance of shell debris. Unexpectedly, no systematic negative correlation was found between nutrient elements and grain size, probably because progressive eutrophication caused both higher OM content and coarser grain size due to shell debris, especially in the 1^st^ basin. It is also possible that the coarse fraction was overestimated in some samples due to aggregates formed by OM fibres.

**Fig. 6:**
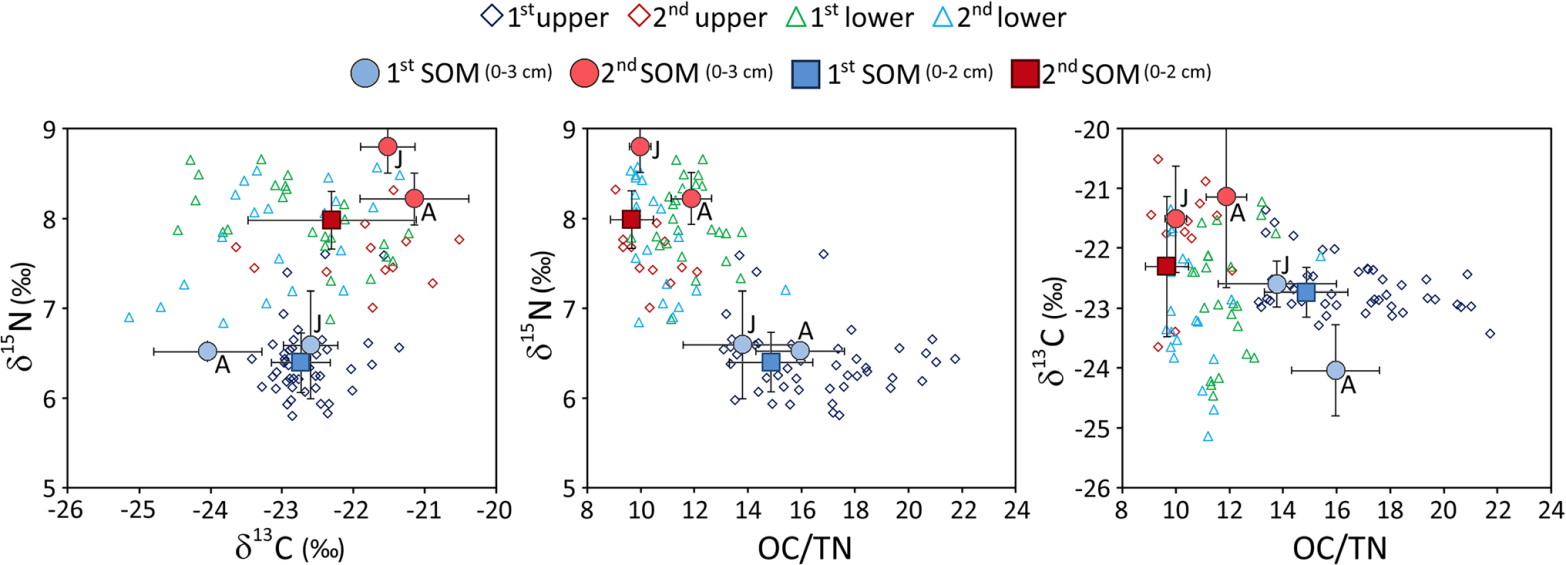
Plots of δ^15^N against δ^13^C, δ^15^N against the OC/TN ratio and δ^13^C against the OC/TN ratio in samples grouped by depth (upper and lower sections) and basin (1^st^ and 2^nd^), as defined in the text. Symbols marking SOM 0–3 cm (sediment organic matter at depths of 0–3 cm) show mean values with standard deviations obtained from surface sediments by Bongiorni et al. (2016) in June 2013 (J) and April (A) 2014 from two sites in the 1^st^ and 2^nd^ basins. Symbols marking SOM 0–2 cm show mean values with standard deviations obtained from surface sediments in this study (sampled in June 2013, 7 sites in the 1^st^ basin and 3 sites in the 2^nd^ basin)

The plot of δ^15^N against δ^13^C showed perfect independence of the two variables for the whole dataset (Fig. 6). Nearly all samples from the upper section of the 1^st^ basin had lower δ^15^N values and a narrower δ^13^C range than other groups. The narrow δ^13^C range during the eutrophic phase can be interpreted as confirming the homogeneity of the sources for the whole 1^st^ basin, characterised by a fairly uniform mixture of OM originating from phytoplankton, macroalgae and terrestrial POM. However, a relatively large range of OC/TN ratios, between 13 and 22 (Fig. 6), suggested that the dominant OM fraction in certain periods was of terrestrial origin. A strong spike of terrestrial POM was recorded in some cores from the 1^st^ basin resulting in C/N ratio of SOM close to 20 (Fig. 5) and dated around 1930–1940 in core 1F. This can possibly be attributed to POM in untreated sewage discharged from industrial, naval and/or urban areas. The proportion of this type of POM gradually decreased leading to the mean C/N ratio of 14.9 in the SOM at surface (Fig. 6).

The isotopic signature of SOM obtained by Bongiorni et al. (2016) from surface sediments (0–3 cm) was consistent with the mean values we obtained (0–2 cm) from cores collected in the 1^st^ (*n* = 7) and 2^nd^ (*n* = 3) basins. Thus, the difference between the sediments of the two basins during the eutrophic phase persisted until recently.

The statistical treatment of data from all cores and groups (upper and lower sections, 1^st^ and 2^nd^ basins) is summarised in Table S4.1 (mean, median, minimum and maximum values for the whole dataset and the subsets) in Supplementary Information. The ratios of the medians of the individual variables are shown in Table 1, with significant differences in bold.

**Table 1:** Ratio of medians of organic carbon (OC), total nitrogen (TN), their atomic ratio, isotopic signatures, carbonate contents (CaCO_3_) and median particle size (D50) in the upper (U) and the lower (L) sections of sediments in the 1^st^ and the 2^nd^ basins of the Mar Piccolo. Significant differences in bold (Mann–Whitney test at *p* = 0.05)

Ratio	OC	δ^13^C	TN	δ^15^N	OC/TN	CaCO_3_	D(50)
U/L	**2.06**	0.99	**1.51**	**0.83**	**1.38**	**1.11**	**1.31**
U 1^st^ /L 1^st^	**1.57**	1.01	1.12	**0.80**	**1.42**	1.01	1.19
U 2^nd^ /L 2^nd^	**2.49**	**0.93**	**2.68**	0.94	1.04	1.01	0.92
U 1^st^/U 2^nd^	1.12	**1.06**	**0.66**	**0.83**	**1.58**	**1.28**	**2.14**
L 1^st^/L 2^nd^	**1.78**	0.97	**1.57**	0.98	**1.15**	**1.28**	**1.66**

**Fig. 7:**
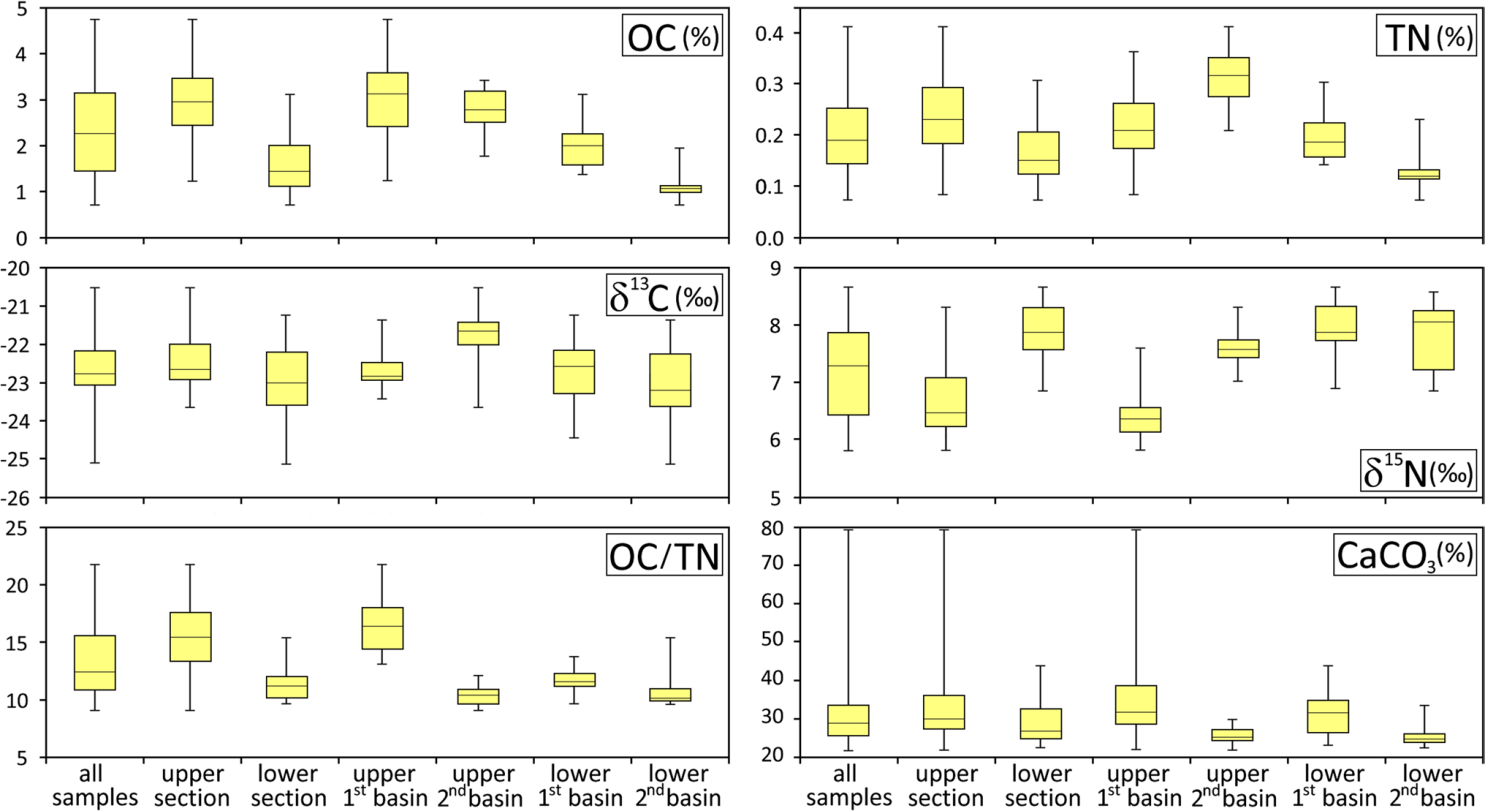
Box plots of measured variables showing medians, quartiles, minima and maxima of measured parameters for all samples, upper and lower sections of sediments (as defined in the text) and upper and lower sections of sediments in the 1^st^ and 2^nd^ basins of the Mar Piccolo. Numerical statistics are shown in Table S4.1 in Supplementary Information

In both basins, OC content was significantly higher in the upper section of sediments than the lower section. Sediments from the lower section of the 1^st^ basin were richer in OC than the lower section of the 2^nd^ basin, but there was no significant difference between the basins in the upper sections (Fig. 7, Table 1).

The TN content in the 2^nd^ basin was 2.7 times higher in the upper section than the lower section, while in the 1^st^ basin there was no significant difference between TN content in the upper and lower sections (Fig. 7, Table 1). Although the sediments in the lower section of the 1^st^ basin were richer in TN than the lower section of the 2^nd^ basin, the opposite was true for the upper sections. Consequently, the OC/TN ratio in the 1^st^ basin was significantly higher in the upper section than the lower section, while there was no significant difference in the 2^nd^ basin.

The contrast in terms of δ^13^C and δ^15^N between cores 1F and 2C during the eutrophic phase can be generalised to the whole of the 1^st^ and 2^nd^ basins (Fig. 7). There were only minor differences in δ^13^C signatures between the subsets. The upper section of sediment in the 2^nd^ basin has slightly (but significantly) less negative values, suggesting a relatively higher contribution of phytoplankton and/or macroalgae to the buried OC than the upper sections of sediments in the 1^st^ basin. Indeed, the satellite mapping invariably shows a higher concentration of phytoplankton during summer blooms (Borfecchia et al. 2019) and denser green macroalgae coverage (Cibic et al. 2016) in the 2^nd^ basin.

In contrast to δ^13^C, δ^15^N showed considerable variability between groups. The strongest difference was a δ^15^N value in the upper sections (6.4‰) 20% below that of the lower sections (7.9‰) in the 1^st^ basin.

CaCO_3_ content was 28% higher in the 1^st^ basin than the 2^nd^ basin, in both the upper and lower sections. This was due to more abundant shell fragments, especially near the *Canale navigabile*.

### Estimation of the OC burial rate

Judging by the fairly consistent evolution of the various markers with depth in most of the sediment cores, it can reasonably be assumed that the mean OC content in the sediments of each basin, multiplied by the MAR measured in cores 1F and 2C, approximates the OC burial rate in the two basins of the Mar Piccolo in different periods. Table 2 shows the values for the two basins, along with the estimated uncertainty.

**Table 2:** Mean content of organic carbon (OC) in sediments in two basins of the Mar Piccolo and the estimated burial rates in surface sediments, and during eutrophic and oligotrophic phases. MAR, mass accumulation rates of dry sediments. Assuming the same MAR for all sites in each basin and uncertainties of means of OC contents at 1 standard deviations (*σ*), the propagated uncertainties OC burial (CV) are between 25 and 33%

1^st^ basin		Mean OC		MAR		OC burial rate		
Approximative period		mg g^−1^	σ	g cm^−2^y^−1^	σ	g m^−2^y^−1^	σ	CV
2005–2013	Recent 0–2 cm	33	10	0.15	0.01	49	15	31%
≈1930–2013	Eutrophic	30	9	0.15	0.01	45	14	30%
Before ≈1930	Oligotrophic	20	5	0.15	0.01	30	7	25%
2^nd^ basin		Mean OC		MAR		OC burial rate		
Approximative period		mg g^−1^	σ	g cm^−2^y^−1^	σ	g m^−2^y^−1^	σ	CV
2005–2013	Recent 0–2 cm	30	6	0.17	0.04	50	16	32%
≈1930–2013	Eutrophic	28	5	0.17	0.04	47	29	29%
Before ≈1930	Oligotrophic	11	3	0.17	0.04	19	6	33%

An alternative method of calculating the OC burial rate for the eutrophic period was based on the assumption that the onset of eutrophic conditions (marked by the borderline depth in the dated cores 1F and 2C for the 1^st^ and 2^nd^ basin respectively) was synchronous in all cores within each basin. The mean OC content in the upper (eutrophic) section was multiplied by the estimated MAR at each site and the resulting burial rate was averaged for the cores in each basin. For both basins, the results differ from those given in Table 2 by about 20%, albeit in opposite directions, but the mean of 49 g m^−2^ y^−1^ is very similar to what was obtained with the first method.

The OC burial rate in the Mar Piccolo during the eutrophic period was similar to the estimated median OC burial rate in the world's lagoons of 41.4 g m^−2^ y^−1^ (Wilkinson et al. 2018), although during the preceding oligotrophic period it amounted to only 40–60% of the burial rate during the eutrophic period.

The approximate value of 46 g of OC m^−2^ y^−1^ buried in sediment during the eutrophic period corresponds to about 952 t y^−1^ for the total surface area of Mar Piccolo (20.7 km^2^). Extrapolation should be viewed with caution however, as nearly half of the Mar Piccolo surface area is used for mussel production and thus was not sampled, because of restricted access. The bacteriological study by Zaccone et al. (2005) suggested that the OM content of sediments in the mussel cultivation area was higher than in other parts of the Mar Piccolo basins and thus the calculated OC burial rate is probably underestimated.

Mussel farming may strongly interact with the host ecosystem in various ways (Prins et al. 1997), especially in semi-enclosed basins with poor flushing and high-density aquaculture (Bouwman et al. 2011). This is the case of the Mar Piccolo due to its relatively long water renewal time (De Pascalis et al. 2016) and the large proportion of area dedicated to mussel farming (Massarelli et al. 2021). Mussels remove the seston, assimilate a part of the POM and release its fraction as particulate and dissolved nutrients into the water (Bouwman et al. 2011). Particulate OC, N, P and mineral particles excreted as faeces and pseudofaeces accumulate in underlying sediments. In sediments under mussel culture, the OC concentration and the burial rate are expected to be higher, which will lead to a net decrease in sedimentation of organic material on a basin scale (Timmermann et al. 2019).

The OC burial rate at mussel farm sites depends on a multitude of factors such as the culture method (physical structures, its density), hydrology (local current speed modified by the physical structures, sediment resuspension capacity), rate of OC release by mineralisation of OM and other (McKindsey et al. 2011). Numerous studies have shown OC enrichment factors in sediments under mussel culture in comparison to the reference sites, varying from 1.1 to 2.6 (McKindsey et al. 2011). The variability may be even higher when comparing OC burial fluxes, due to generally enhanced sedimentation rates under mussel culture. It is therefore not possible to assess the rate of OC burial in the Mar Piccolo mussel farming area without a specifically oriented study.

Calculations by Giordano et al. (2019) showed that under the best physiological conditions, as assessed by condition index analysis from 1974 to 2002–2004, mussels cultured in Mar Piccolo used about 1250 t y^−1^ carbon to grow, comparable to the OC burial rate in sediments outside mussel culture area during the eutrophic period.

## Conclusions

Statistical treatment of the subsets of collected data confirmed and reinforced the observations based on the examination of dated cores 1F and 2C. In the sediments of both basins of the Mar Piccolo, beginning around 1928–1935 and culminating between 1960 and 1970, enhanced accumulation and storage of OC was evidenced. This was due to eutrophication and, especially in the 1^st^ basin, a large load of POM derived from untreated sewage.

The increase in sediment OC content mirrored population growth in Taranto, although OC content levelled off a decade earlier than the population. This may be attributed to some physico-chemical factors limiting phytoplankton production, but more likely to the increase in mussel production and consequent increased grazing.

Before eutrophication, the 1^st^ basin received or produced more OM than the 2^nd^, but there were no notable differences in the isotopic signals of OC and TN, suggesting similar sources for both basins. In the eutrophic period, a clear difference in the type of SOM appeared. In the 2^nd^ basin, the OC/TN ratio and δ^15^N did not change much, while in the 1^st^ basin, the OC/TN ratio increased and δ^15^N decreased. This evolution was probably due to the greater impact of sewage and industrial pollution in the 1^st^ basin and the progressive growth of mussel production in the 2^nd^. The less negative δ^13^C of SOM in the upper section of the 2^nd^ basin suggests that the increase in OC content was due to in situ biomass production. The narrower range of δ^13^C (on average unchanged) and higher OC/TN ratio in the upper section of the 1^st^ basin suggested increased input of external OM sources, with a notable contribution of sewage-derived POM. Severe chemical pollution concomitant with eutrophication caused the degradation of ecosystem functioning, especially in the 1^st^ basin.

To improve the interpretation of the history of eutrophication, the variations in OC and TN content and their isotopic signatures should be determined with better temporal resolution and supplemented by examination of other proxies, such as the presence of ostracods as well as pigment and lipid biomarkers.

The OC burial rate in Mar Piccolo sediments during the eutrophic phase was close to the global median value estimated for lagoon sediments, but about twice the rate recorded in the preceding oligotrophic phase. As eutrophication affects many lagoons and coastal seas worldwide, the resulting enhanced OC burial rate may be of importance concerning the global CO_2_ budget over the past century.

## Supplementary Information

Below is the link to the electronic supplementary material.

Supplementary file1 (DOCX 4.05 MB)

## Data Availability

The datasets used or analysed during the current study are available from the corresponding author on reasonable request.
