# Supplementary material for: Eutrophication history and organic carbon burial rate recorded in sediment cores from the Mar Piccolo of Taranto (Italy)
Source: Environ Sci Pollut Res Int. 2023 Mar 16;30(19):56713–30. doi: 10.1007/s11356-023-26317-6 (PMC10121521; doi:10.1007/s11356-023-26317-6)
Supplement: Supplementary file 1 — Supplementary file1 (DOCX 4.05 MB) [file 11356_2023_26317_MOESM1_ESM.docx]

***Environmental Science and Pollution Research***

**Eutrophication history and organic carbon burial rate recorded in sediment cores from the Mar Piccolo of Taranto (Italy).**

Janusz Dominik^1^, Simone Leoni^1^, Daniele Cassin^1^, Irene Guarneri^1^, Luca Giorgio Bellucci^2^ and Roberto Zonta^1*^

^1^ Istituto di Scienze Marine, Consiglio Nazionale delle Ricerche (ISMAR-CNR), Castello 2737/F, 30122 Venezia, Italy
^2^ Istituto di Scienze Marine, Consiglio Nazionale delle Ricerche (ISMAR-CNR), Via Gobetti 101, 40129 Bologna, Italy

* corresponding author: r.zonta@ismar.cnr.it; +39 339 2006972

# Supplementary Information

# S1. In-situ dry density of sediments ρ^s^

In all subsamples (2 cm thickness) water content (W_c_) was determined as (wet weight – dry weight)/wet weight. From the W_c_ and the assumed mean density of sediment particles, the in-situ dry density was calculated (ρ^s^ in g cm^-3^). The calculation assumed the following specific density of the components: organic matter (OM) = 1.25, carbonates (CaCO_3_) = 2.8 and the remaining particles (silicate and aluminium-silicate) = 2.6 g cm^-3^. OM was obtained as organic carbon (OC) * 1.7 and CaCO_3_ as inorganic carbon (IC) * 8.3.

Mean CT numbers (AIP) were measured for the sampled intervals in 2 cm depth sections. The linear regression between ρ^s^ and the CT number for 100 samples is shown in Fig. S1.1.


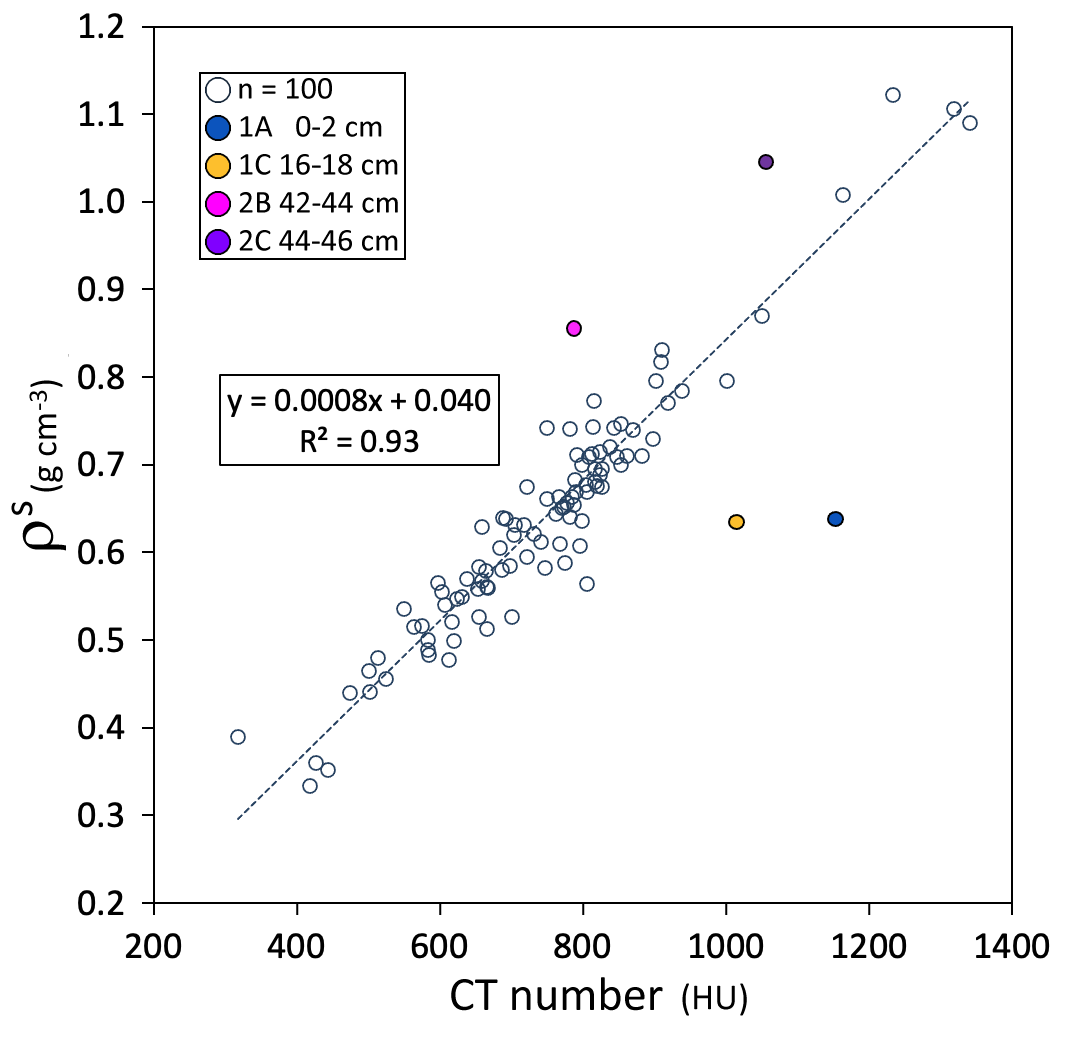


Fig. S1.1. Linear regression of the CT number (AIP setting, 2 cm-thick samples) against the calculated in-situ dry density (ρ^s^). Four samples were considered outliers (coloured dots).

Excluding the four outliers, a high regression coefficient (R^2^ = 0.93) was obtained. Outliers were identified as samples with a poor correspondence between subsamples used for water content determination and the volume used in the CT measurements. In surface sample 1A 0-2 cm, pore water content was overestimated, probably due to the inclusion of overlying water in the sample; in sample 1C 16-19 cm a large shell was removed before subsampling; samples 2B 42-44 cm and 2C 44-46 cm were not homogeneous because of the presence of high- and low-density layers (see Fig. 4 in the main text).

Using the regression from Fig S1.1, continuous profiles of ρ^s^ with depth (0-50 cm, 2 cm resolution) were obtained for all cores. These are shown in Fig. S1.2 along with CT images of cores collected in the 1^st^ basin (for profiles of cores from the 2^nd^ basin, see Fig. 4 in the main text).


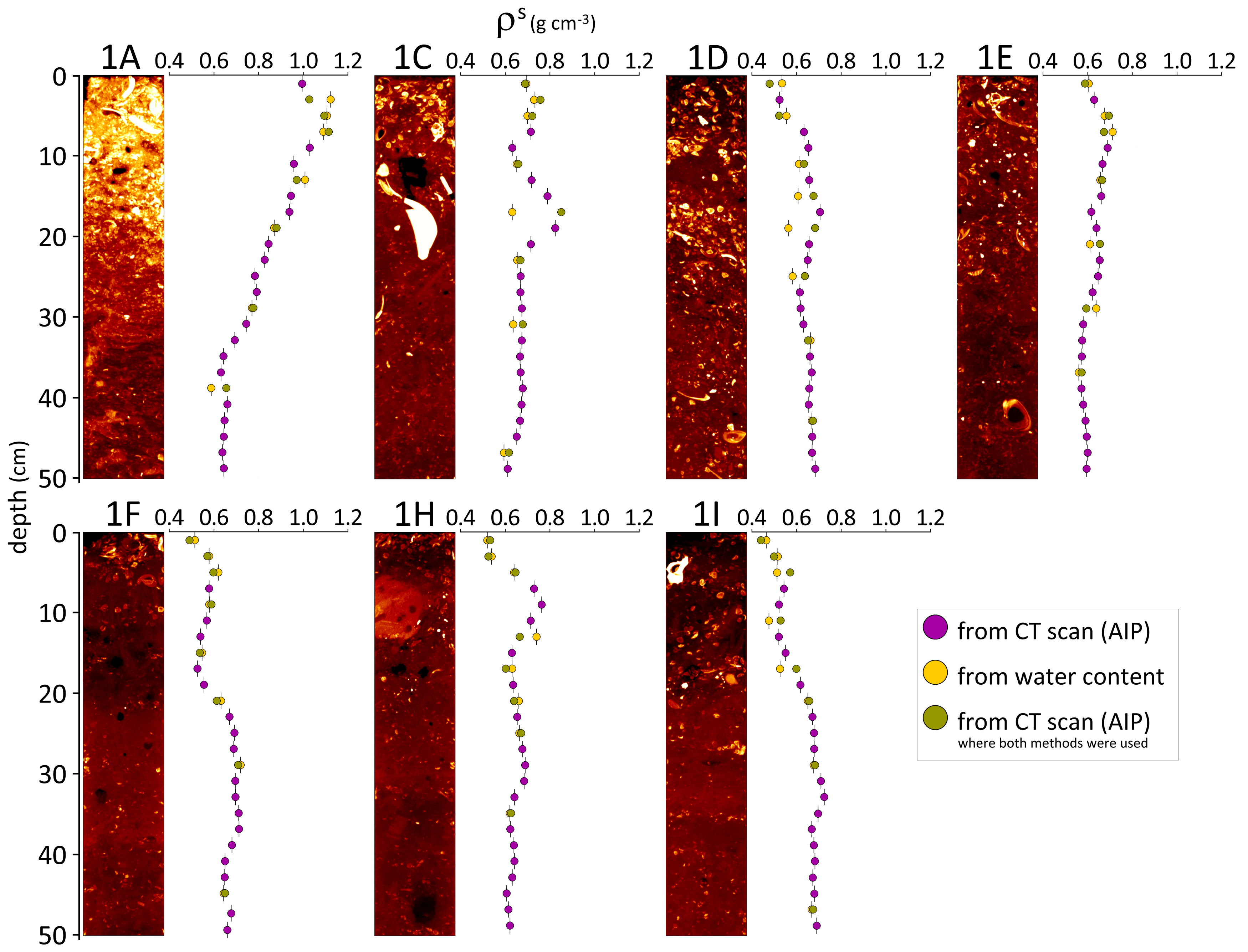


Fig S1.2. CT images in MIP (Maximum Intensity Projection) of the upper 50 cm of cores collected in the 1^st^ basin along with in-situ dry density (ρ^s^) profiles with depth. Darker colours correspond to lower density sections i.e. volumes with high W_c_. Lighter colours correspond to higher density sections, i.e. objects or sections with low W_c_. Green circles show values calculated from the CT number (AIP setting) for samples at depths where in situ dry density was also calculated from W_c_ to show the differences resulting from sample heterogeneity (usually large shells or shell fragments). Figure 4 in the main text shows the corresponding sediment profiles in cores from the 2^nd^ basin.

**S2. Sediment accumulation rate determined with the ^210^Pb method.**

In the cores for which ^210^Pb dating was attempted (1F, 2C), the in-situ density of non-sampled intervals was obtained from a separate regression between the CT number and in-situ density (y = 0.00096x - 0.063) with R^2^ = 0.91.


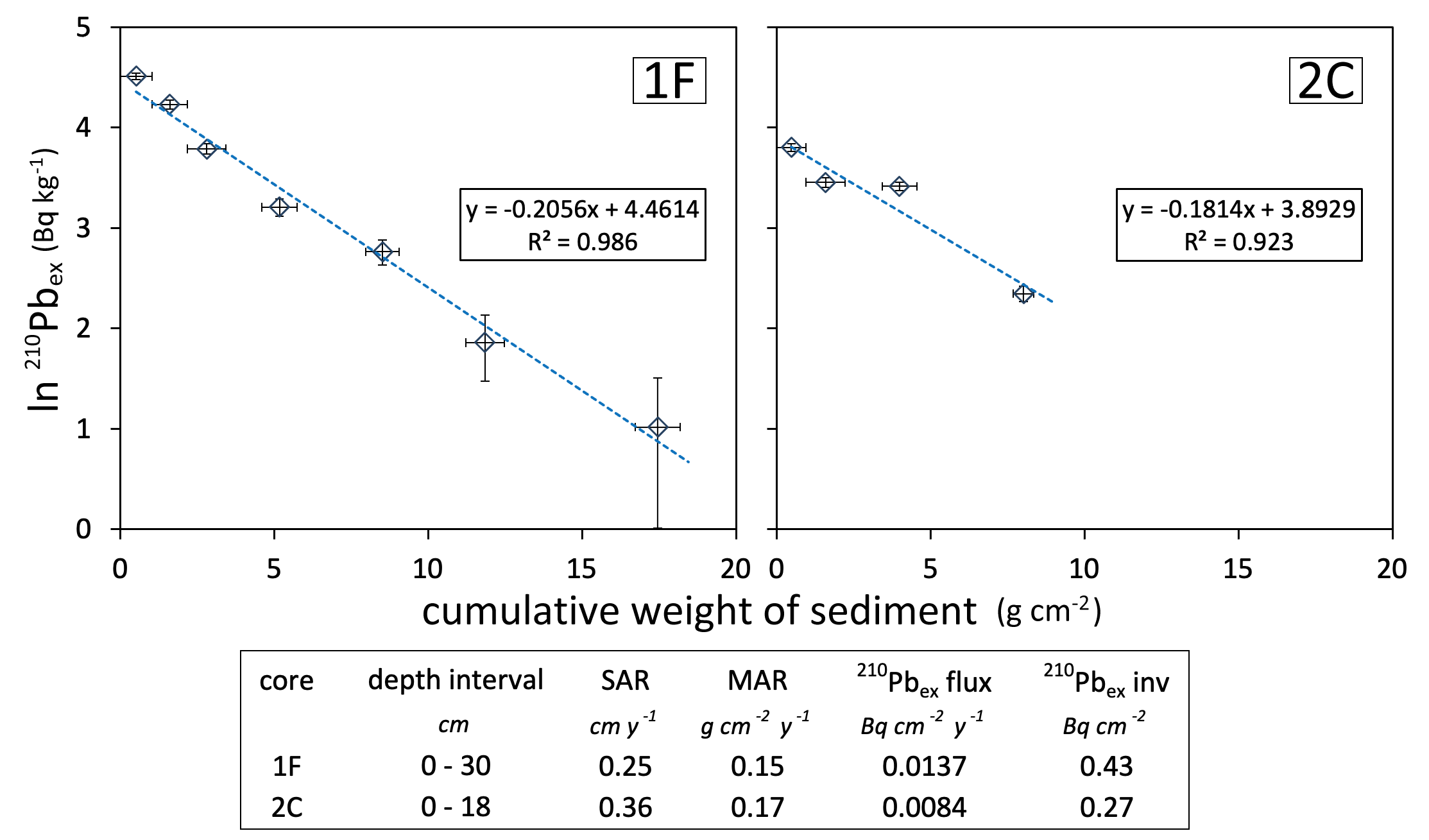


Fig. S2. Plots of ln ^210^Pb_ex_ against the cumulative weight of dry sediment in cores from the 1^st^ basin (1F) and 2^nd^ basin (2C). The resulting sediment mass accumulation rate in g cm^-2^ y^-1^ (MAR) and the mean sediment accumulation rate with depth (SAR, cm y^-1^) are shown in the table below the plots, along with ^210^Pb_ex_ fluxes and inventories.

**S3. Magnetic susceptibility (χ)**


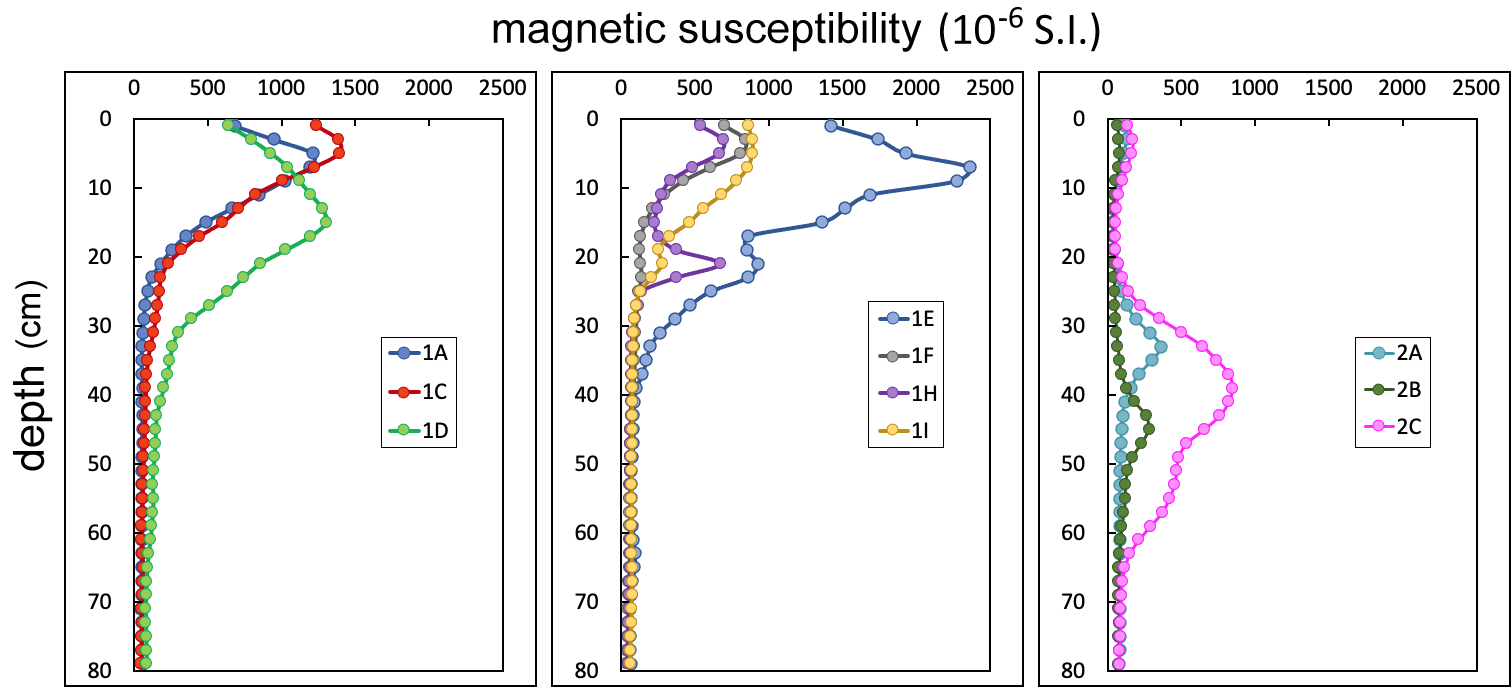


Fig. S3.1 Magnetic susceptibility (χ) profiles in sediment cores from the 1^st^ (1A-1I) and 2^nd^ (2A-2C) basins of the Mar Piccolo.


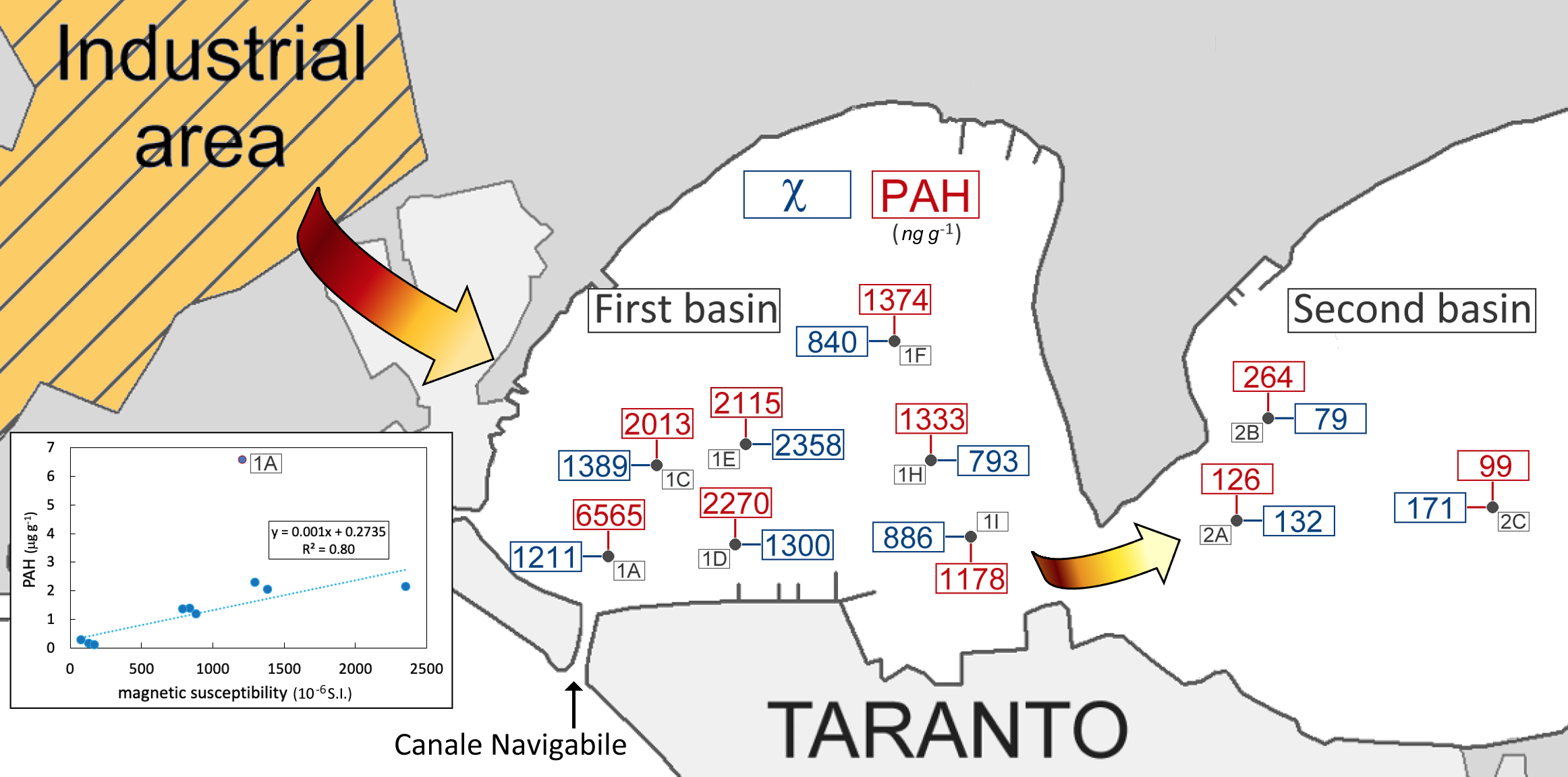


Fig S3.2 Peak magnetic susceptibility (χ, 10^-6^, SI) in the upper sections of sediment cores, showing a decreasing trend from W to E (in blue). Concentrations of total polyaromatic hydrocarbons (ΣPAHs, ng g^-1^, in red) in sediments at the depth of the χ peaks show a decreasing trend with distance from the major industrial plants (our unpublished data). The plot on the left shows the linear regression between peak χ values and ΣPAHs at the same depths. Core 1A is excluded from the regression because its ΣPAH content was influenced by strong boat traffic in the *Canale Navigabile*.

**S4. Descriptive statistics of measured variables in the Mar Piccolo sediments**

Descriptive statistics (Table S4.1) were calculated for samples from all cores, for the upper and lower sections of all cores, and for the lower and upper sections of cores from each basin separately. Two samples not belonging to recent Mar Piccolo sediments sampled in the bottom of cores 1C and 1E were excluded from the statistics.

Since most cores show gradients of examined variables at depths of between 20 and 35 cm, the cores were divided into two sections (Table S4.2). The borderline depth was determined for each core based mainly on OC, C/N and δ^15^N, as these variables usually showed a clear-cut gradient.

Table S4.1. Descriptive statistics for nutrient elements, their isotopic signatures, carbonate content and median grain size for all sediment samples, upper and lower sections (as defined in the text) and upper and lower sections of cores from each basin separately, in the 1^st^ and 2^nd^ basins of the Mar Piccolo.

|  |  | OC % | δ^13^C ‰ | TN % | δ^15^N ‰ | OC/TN^b^ | CaCO_3_^c^ % | D(50) µm^e^ |
| --- | --- | --- | --- | --- | --- | --- | --- | --- |
| 10 cores, | mean | 2.34±0.99 | -22.7±0.8 | 0.20±0.08 | 7.2±0.8 | 13.4±3.3 | 32±10 | 15±31 |
| all depth, n = 102^a^ | median | 2.27 | -22.8 | 0.19 | 7.3 | 12.5 | 29 | 7.5 |
|  | max | 4.74 | -20.5 | 0.41 | 8.7 | 21.7 | 79 | 198 |
|  | min | 0.72 | -25.1 | 0.07 | 5.8 | 9.1 | 22 | 4.5 |
|  | normal^d^ | no | yes | no | no | no | no | no |
|  |  |  |  |  |  |  |  |  |
| 10 cores | mean | 2.95±0.82 | -22.5±0.7 | 0.24±0.08 | 6.7±0.6 | 15.2±3.4 | 34±12 | 21±43 |
| upper section, n=56 | median | 2.97 | -22.7 | 0.23 | 6.5 | 15.4 | 30 | 8.5 |
|  | max | 4.74 | -20.5 | 0.41 | 8.3 | 21.7 | 79 | 198 |
|  | min | 1.24 | -23.6 | 0.08 | 5.8 | 9.1 | 22 | 4.5 |
|  | normal | yes | no | yes | no | yes | no | no |
|  |  |  |  |  |  |  |  |  |
| 10 cores, | mean | 1.60±0.58 | -22.9±1.0 | 0.16±0.05 | 7.9±0.5 | 11.3±1.3 | 29±5 | 7.8±2.9 |
| lower section, n=46^a^ | median | 1.44 | -22.9 | 0.15 | 7.9 | 11.2 | 27 | 6.4 |
|  | max | 3.11 | -21.23 | 0.30 | 8.66 | 15.41 | 43.80 | 16.7 |
|  | min | 0.72 | -25.14 | 0.07 | 6.84 | 9.64 | 22.41 | 4.5 |
|  | normal | no | yes | no | no | no | no | no |
|  |  |  |  |  |  |  |  |  |
| 1^st^ basin - 7 cores, | mean | 3.00±0.89 | -22.7±0.4 | 0.21±0.07 | 6.4±0.4 | 16.6±2.4 | 36±13 | 25±44 |
| upper section, n= 44 | median | 3.12 | -22.8 | 0.21 | 6.3 | 16.4 | 32 | 10.6 |
|  | max | 4.73 | -21.4 | 0.36 | 7.6 | 21.7 | 79 | 198 |
|  | min | 1.24 | -23.4 | 0.08 | 5.8 | 13.1 | 22 | 5.1 |
|  | normal | yes | no | yes | no | yes | no | no |
|  |  |  |  |  |  |  |  |  |
| 2^nd^ basin - 3 cores, | mean | 2.77±0.48 | -21.8±0.9 | 0.31±0.07 | 7.6±0.3 | 10.3±0.9 | 25±3 | 5.1±0.5 |
| upper section, n=12 | median | 2.78 | -21.6 | 0.32 | 7.6 | 10.4 | 25 | 5,0 |
|  | max | 3.43 | -20.5 | 0.41 | 8.3 | 12.1 | 30 | 5.9 |
|  | min | 1.78 | -23.6 | 0.21 | 7.0 | 9.1 | 22 | 4.5 |
|  | normal | yes | yes | yes | yes | yes | yes | yes |
|  |  |  |  |  |  |  |  |  |
| 1^st^ basin - 7 cores, | mean | 1.99±0.48 | -22.8±0.9 | 0.20±0.05 | 7.9±0.4 | 11.8±1.0 | 31±5 | 9.1±2.8 |
| lower section, n=25 | median | 1.99 | -22.6 | 0.19 | 7.9 | 11.6 | 32 | 9.0 |
|  | max | 3.11 | -21.2 | 0.30 | 8.7 | 13.8 | 44 | 16.7 |
|  | min | 1.38 | -24.5 | 0.14 | 6.9 | 9.7 | 23 | 5.0 |
|  | normal | yes | yes | no | yes | yes | yes | yes |
|  |  |  |  |  |  |  |  |  |
| 2^nd^ basin - 3 cores, | mean | 1.14±0.27 | -23.1±1.0 | 0.13±0.03 | 7.8±0.6 | 10.6±1.3 | 25±3 | 6.3±2.5 |
| lower section, n=21 | median | 1.12 | -23.2 | 0.12 | 8.1 | 10.0 | 25 | 5.4 |
|  | max | 1.94 | -21.3 | 0.23 | 8.6 | 15.4 | 34 | 15.0 |
|  | min | 0.72 | -25.1 | 0.07 | 6.8 | 9.6 | 22 | 4.5 |
|  | normal | no | yes | no | no | no | no | no |

^a^ two samples from the bottom of cores 1C (114 cm) and 1E (97 cm) are omitted because they belong to a different sedimentary unit; ^b^ atomic ratio; ^c^ calculated from inorganic carbon content; ^d^ distribution normality test Shapiro-Wilk at p =0.05; ^e^ median particle-size

Table S4.2 Division of the cores into upper (0-borderline depth) and lower (below borderline depth) sections.

| core | borderline depth (cm) | sample depth above (cm) | sample depth below (cm) |
| --- | --- | --- | --- |
| 1A | 34 | 29 | 39 |
| 1C | 27 | 23 | 31 |
| 1D | 38 | 27 | 43 |
| 1E | 33 | 29 | 37 |
| 1F | 25 | 21 | 29 |
| 1H | 23 | 21 | 25 |
| 1I | 25 | 21 | 29 |
| 2A | 22 | 15 | 29 |
| 2B | 23 | 15 | 31 |
| 2C | 25 | 17 | 33 |


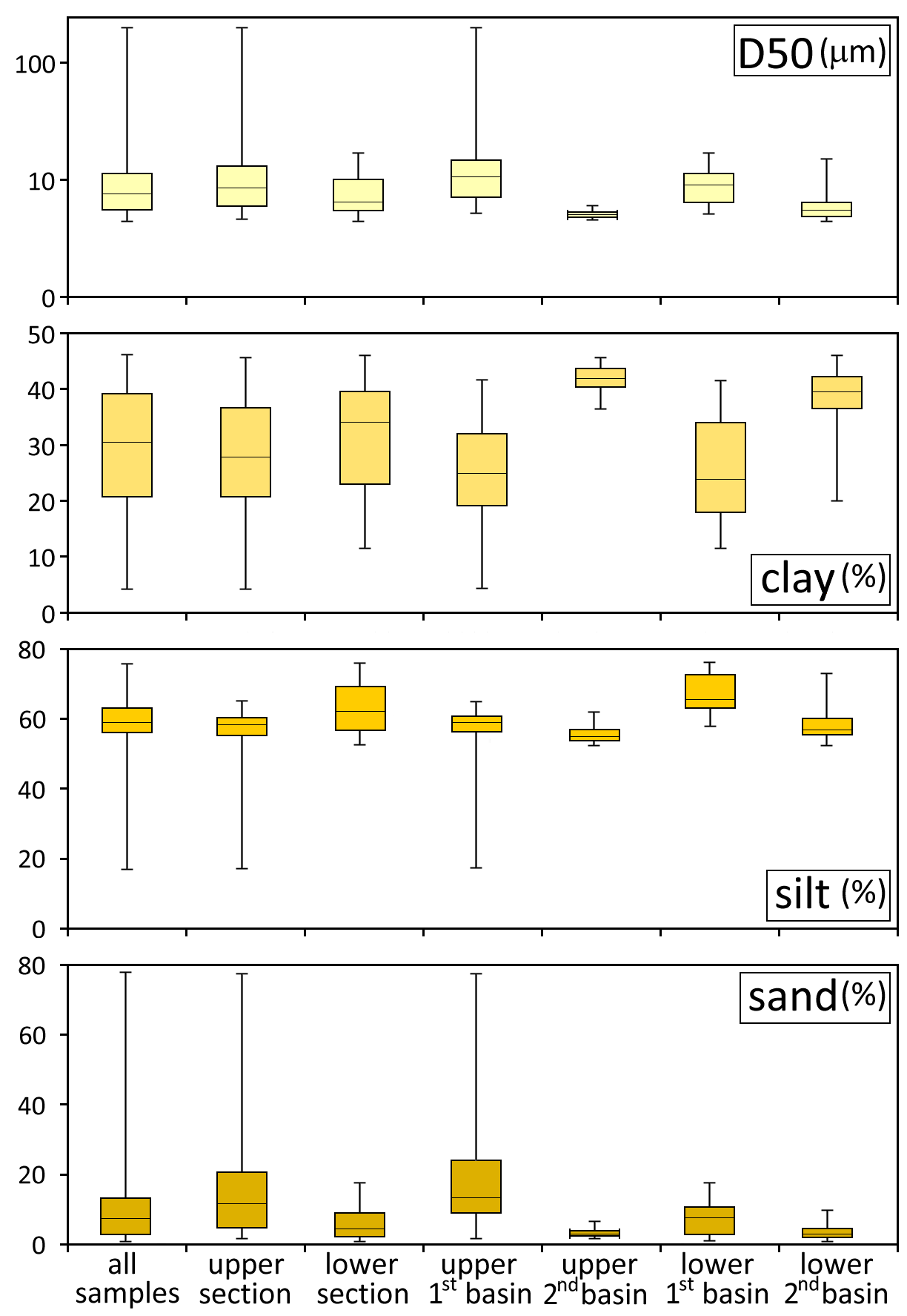


Fig S4. Box plots of median grain size (log scale) and percentage of grain-size fractions showing medians, quartiles, minima and maxima for all samples, upper and lower sections of sediments (as defined in the text), in the 1^st^ and 2^nd^ basins of the Mar Piccolo. The numerical values of the median particle size (D50) are shown in Table S4.1.
